# Current Strategies and Future Directions of Wearable Biosensors for Measuring Stress Biochemical Markers for Neuropsychiatric Applications

**DOI:** 10.1002/advs.202411339

**Published:** 2024-12-17

**Authors:** Zach Sheffield, Priyanka Paul, Shraddha Krishnakumar, Dipanjan Pan

**Affiliations:** ^1^ Huck Institutes of the Life Sciences The Pennsylvania State University State College PA 16802 USA; ^2^ Department of Nuclear Engineering The Pennsylvania State University State College PA 16802 USA; ^3^ The Center for Advanced Sensing Technology University of Maryland – Baltimore County Baltimore MD 21250 USA; ^4^ Chemical, Biochemical and Environmental Engineering Department University of Maryland – Baltimore County Baltimore MD 21250 USA; ^5^ Department of Pediatrics University of Maryland Baltimore School of Medicine Baltimore MD 21201 USA

**Keywords:** cortisol, multiplex, neuropsychiatric, peripheral biofluids, stress, wearable biosensors

## Abstract

Most wearable biosensors aimed at capturing psychological state target stress biomarkers in the form of physical symptoms that can correlate with dysfunction in the central nervous system (CNS). However, such markers lack the specificity needed for diagnostic or preventative applications. Wearable biochemical sensors (WBSs) have the potential to fill this gap, however, the technology is still in its infancy. Most WBSs proposed thus far target cortisol. Although cortisol detection is demonstrated as a viable method for approximating the extent and severity of psychological stress, the hormone also lacks specificity. Multiplex WBSs that simultaneously target cortisol alongside other viable stress‐related biochemical markers (SBMs) can prove to be indispensable for understanding how psychological stress contributes to the pathophysiology of neuropsychiatric illnesses (NPIs) and, thus, lead to the discovery of new biomarkers and more objective clinical tools. However, none target more than one SBM implicated in NPIs. Till this review, cortisol's connection to dysfunctions in the CNS, to other SBMs, and their implication in various NPIs has not been discussed in the context of developing WBS technology. As such, this review is meant to inform the biosensing and neuropsychiatric communities of viable future directions and possible challenges for WBS technology for neuropsychiatric applications.

## Graphical abstract

This review aims to provide much needed context for the monitoring stress‐related biochemical markers in peripheral biofluids with wearable biosensors for neuropsychiatric applications. In doing so, cortisol's involvement in psychological stress is explained, discussed targeting other stress‐related biomarkers, the need for multiplex devices for neuropsychiatric applications, and the current state and future challenges of wearable biosensing technology for such applications.

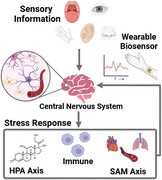

## 1 Introduction

Stress, in the field of neuroendocrinology, refers to any external or internal stimuli that perturb the body from a state of homeostasis. Societal perception of stress is often negative. It is seen as something which should be avoided or remedied. On the contrary, normally, stress plays a fundamental role in physical and neurological adaptation.^[^ ^1^, ^2^ ^]^ When we encounter a perceived stressor, our body responds by secreting “stress mediators” that serve to return itself to a state of homeostasis. Ideally, the new homeostatic state will be better able to counteract any negative, bodily effects if the same stressor were applied again. This is how our bodies learn to adapt and overcome. However, the negative connotation of stress is not unfounded, especially regarding how it can be detrimental to the brain. Instances of repeated acute or chronic mild psychological stress can be particularly harmful to the brain and can contribute to the pathophysiology of neuropsychiatric illnesses (NPIs). This is especially true for certain subsets of the world population, such as children and those genetically predisposed to be vulnerable to stress.^[^ ^3^, ^4^, ^5^, ^6^ ^]^

An individual suffering from an NPI, or is at high risk of developing an NPI, would respond to stressors differently than individuals without these illnesses. Consequently, one would expect there to be downstream changes, defined by biomarkers, in the physiology of connected peripheral systems that are representative of these individuals’ neurological state. Indeed, there are stress‐related biochemical markers (SBMs) that have been confirmed and correlated with pathways involved in NPIs, however, their current clinical utility remains limited if nonexistent.^[^ ^7^ ^]^ This is in part due to the heterogeneity of most NPIs, their time and context dependent nature, and the connected complexity of the stress response. All SBMs are not specific to one type of illness, whether it be neurological, psychological, or physical because, even though different stressors may be processed by different pathways in the brain, all pathways converge at the hypothalamus, which ultimately controls the stress response.

Wearable biochemical sensors (WBSs) are a subclass of point‐of‐care (POC) devices that can enable the real‐time, continuous detection of biochemicals in peripheral biofluids. Since the success of the continuous glucose monitor, WBSs have been a hot topic because they are an achievable route to more personalized healthcare. For NPIs, WBSs are particularly relevant as they potentially enable healthcare providers with an objective way to continuously monitor patients outside the context of a clinical setting or enable patients to self‐monitor.^[^ ^8^, ^9^ ^]^ So far, wearable biosensors that measure physical stress markers have found significant academic success as they have been used to establish measures of markers like heart‐rate variability (HRV), respiration rate, and skin conductivity as symptomatic of some psychiatric illnesses.^[^ ^9^ ^]^ Such devices have been demonstrated as potential tools for patients suffering from NPIs. However, like SBMs, these physical markers are not specific to psychiatric illnesses. Moreso, they can be viewed as symptomatic of general dysfunction of the autonomic nervous system (ANS) and are associated more with short‐lived responses to acute stressors. Therefore, they provide little to no new information about the cause of the symptoms that could not already be obtained through more conventional methods.

Since the publication of Parlak et al.'s seminal paper, there has been an uptick in papers demonstrating WBSs for the detection or continuous monitoring of SBMs in peripheral biofluids.^[^ ^10^ ^]^ However, aside from some exceptions,^[^ ^11^, ^12^, ^13^, ^14^, ^15^ ^]^ the wearable biosensor literature rarely contextualizes how monitoring peripheral SBMs could potentially help in neuropsychiatric healthcare or how the shear complexity of NPIs is a substantial, yet arguably surmountable, challenge for developing the technology. This is especially true for the hormone, cortisol, whose role in neuropsychiatric illnesses, outside of the hippocampal–pituitary–adrenal (HPA) axis, is rarely clarified.^[^ ^16^, ^17^ ^]^ Cortisol is indeed a reliable measure of general physiological stress,^[^ ^18^ ^]^ and has even been demonstrated as a marker of acute mental stress in real time.^[^ ^11^, ^19^ ^]^ However, changes in cortisol are implicated in most neuropsychiatric illnesses, which may be comorbid with each other, or with physical stressors that also activate the body's stress response.^[^ ^20^ ^]^ Yet, much of the WBS literature is still focused on the detection of cortisol. Consequently, the relevance of the hormone's potential as a biomarker for continuous monitoring applications in neuropsychiatry is being misrepresented, thus simultaneously underselling the importance of the technology while overselling the potential of the hormone.

Cortisol is not the only SBM linked with psychological stress. Of the most common ones, such as inflammatory cytokines or brain‐derived neurotrophic factor (BDNF), cortisol plays a role in their generation in peripheral biofluids through activation of pathways that either start or end in the central nervous system (CNS). Additionally, there are evidence of peripheral concentrations of cortisol changing in response to pharmaceutical treatment of certain psychiatric illnesses. The majority of WBSs in the literature that target SBMs target either cortisol or markers of autonomic dysfunction in sweat. The technological achievements therein demonstrate the growing feasibility in these devices having a significant impact for improving current methodologies in neuropsychiatric healthcare. However, considering the complexity of NPIs and the existence of a myriad of SBMs, a greater emphasis on the multiplex detection of a diverse set of markers is needed. This review is meant to provide a comprehensive overview of the current state of WBSs for SBMs within the context of what is currently known about psychological stress in relationship to NPIs. In this way, we hope to clarify possible misconceptions in the wearable biosensing community regarding stress and highlight under investigated areas, potential challenges, and future directions for their development as ancillary tools in neuropsychiatric healthcare and science.

## 2 Neuromodulatory Systems, Stress, and Their Connection to Peripheral Systems

**Figure 1:**
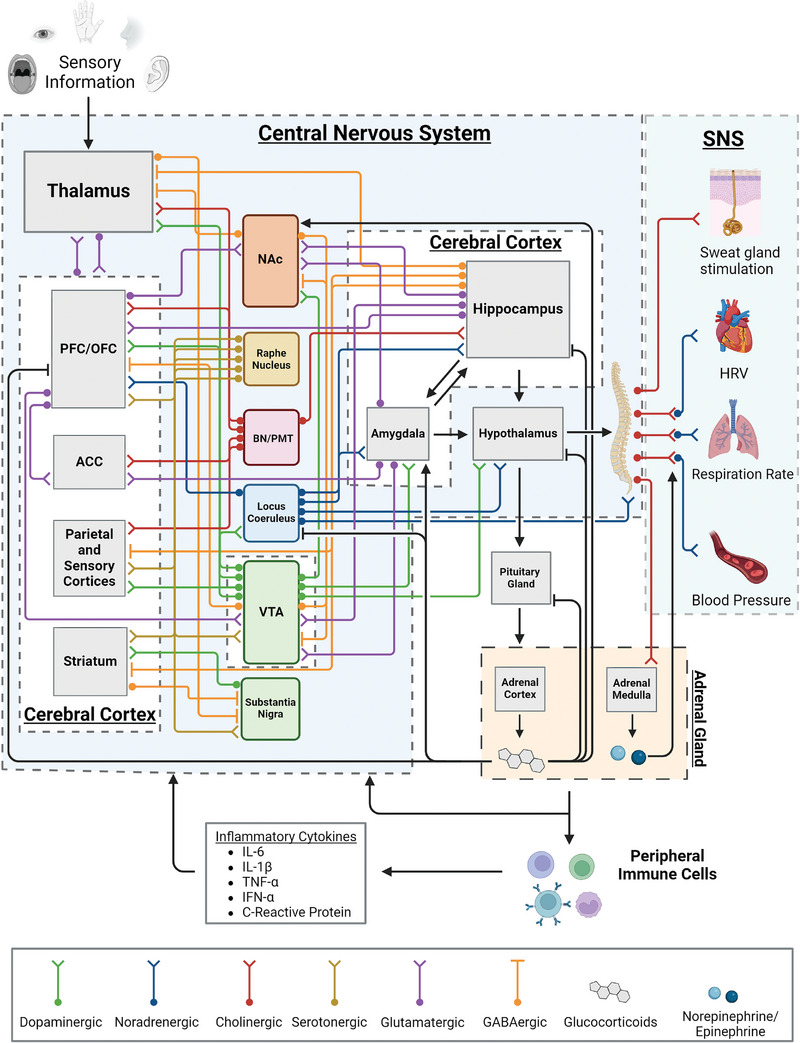
An illustration depicting principal regions of the cerebral cortex affected in NPIs, the known neuronal connections between them, and some of their connections to the peripheral via the HPA axis and SAM axis. The figure does not show the full connectivity between these regions for simplicity. As such, synaptic connections between the principal neurons depicted in the figure and the interneurons and glial cells that reside in the gray matter are not depicted.^[^ ^21^ ^]^ Therefore, the figure is meant to show the complexity of the neuropsychiatric processes that culminate in the body's stress response. Neuronal and peripheral connections were constructed from the following sources.^[^ ^22^, ^23^, ^24^, ^25^, ^26^, ^27^, ^28^ ^]^ Created using Biorender.com.

**Figure** **1** depicts a portion of the intricate network of neural circuitry that controls how we perceive and respond to stressors. The prefrontal cortex (PFC), orbital frontal cortex, anterior cingulate cortex, parietal and sensory cortices, and the striatum are subregions of the cerebral cortex that are not discussed in the context of biochemical sensing of SBMs. Yet, these subregions are responsible for how we process the sensory information that ultimately invokes the stress response in the peripheral. These regions are interconnected with the primary controllers of the stress response, the amygdala, the hippocampus, and the hypothalamus, through neuromodulatory networks composed of neurons that originate from some of these cortical subregions and other nuclei found in the CNS. Studies have drawn correlations between psychological stress and stress related NPIs with losses of functional connectivity, overexcitability, and increases or decreases in volume of some of these cortical subregions. These observations have been evidenced to connect to irregular levels of peripheral SBMs in patients suffering from NPIs. Of special note to the stress response are the prefrontal cortex, the amygdala, and the hippocampus as these regions are found to be critical in humans for the modulation of psychological stress.^[^ ^29^, ^30^ ^]^

### 2.1 Neuromodulatory Systems

The catecholamine‐neuromodulatory systems refer to groups of nuclei found in the central core of the brain, whose axons project throughout the CNS.^[^ ^28^ ^]^ The neurons of each group are responsible for the secretion of a particular neurotransmitter important to the modulation of signal transmissions at synapses in various parts of the brain. Serotonergic neurons project from the raphe nuclei and release serotonin. Adrenergic neurons project from the locus coeruleus and release norepinephrine (aka noradrenaline). Dopaminergic neurons secrete dopamine and project from the ventral tegmental area of the striatum and the substantia nigra. Cholinergic neurons are responsible for the release of acetylcholine and project from the basal nucleus and pontomesencephalo tegmental complex. The interconnections between these nuclei and regions of the cerebral cortex form the basis of our understanding of the neurochemical processes involved in behavior, emotion, fear, learning, memory, mood, attention, cognition, etc.^[^ ^23^ ^]^ First‐line drug therapies for symptom treatment of many neuropsychiatric illnesses such as mood disorders, anxiety disorders, ADHD, bipolar disorder, Parkinson's disease, and post‐traumatic stress disorder (PTSD) will most often target one or more of these neuromodulatory systems.^[^ ^31^ ^]^ There has been consistent evidence of changes in peripheral levels of some SBMs like cytokines,^[^ ^32^ ^]^ cortisol,^[^ ^33^ ^]^ and BDNF,^[^ ^34^ ^]^ following treatment for some psychiatric disorders.

Glutamatergic and GABAergic neurons respectively secrete the amino acid neurotransmitters glutamate and γ‐aminobutyric acid (GABA). Some of their projections into and from the cerebral cortex are depicted in Figure 1. Glutamatergic neurons are the principal excitatory neurons in the cerebral cortex and participate in neural processes involved in signal processing, reward, memory, learning, cognition, and synaptic plasticity and potentiation.^[^ ^22^ ^]^ Glutamate, released at the synaptic cleft, binds with fast‐acting ionotropic receptors that gate the influx of Na^+^ and Ca^2+^ ions needed for action potential generation. GABAergic neurons are the principal inhibitory neurons in the cerebral cortex and participate in many of the same processes as glutamatergic neurons. GABA binds with ionotropic receptors that gate the influx of Cl^−^ ions that modulate and inhibit action potential generation.^[^ ^35^ ^]^ GABA therefore acts to limit the amount of glutamate released at a synapse. Both systems are highly interconnected via their neuronal and glial cell connections and how they participate in each other's metabolisms.^[^ ^36^, ^37^, ^38^ ^]^ Glutamate is of special interest because too much glutamate at the synapse is cytotoxic to brain cells. Imbalances in inhibitory/excitatory function in the cerebral cortex have been linked to common neuropsychiatric conditions including schizophrenia, autism spectrum disorder, MDD, anxiety disorders, epilepsy, and PTSD.^[^ ^35^, ^39^, ^40^, ^41^, ^42^ ^]^ Moreover, as will be touched on in the next section, these modulatory systems play fundamental roles in theoretical pathways of stress dysregulation.

### 2.2 Systems of the Stress Response

The processing of psychological stress, the consequent response to its application, and its subsequent regulation is primarily controlled by the PFC, the amygdala, the hippocampus, the HPA axis, and the SAM axis. Under normal conditions, the PFC enacts top‐down control of the stress response by determining whether sensory, cognitive, or interoceptive (i.e., the perception of bodily sensations) information qualifies as a stressor.^[^ ^20^, ^27^, ^30^, ^43^, ^44^ ^]^ Under acute or chronic stress, control switches to a bottom‐up configuration run by the hippocampus and the amygdala. The hypothalamus acts as a nexus point through which information from the hippocampus and the amygdala passes. The HPA axis and the sympathetic‐adrenal medulla (SAM) axis are responsible for the body's stress response,^[^ ^20^ ^]^ which constitutes the secretion of hormones and neurotransmitters in the peripheral that mediate the genomic, metabolic, and immunological changes that act to bring the body back to homeostasis. The SAM axis is responsible for the initial phase of the stress response, which activates quickly and results in short‐lived symptoms. The HPA axis comprises the second phase of the stress response. It activates slower than the SAM axis and results in longer‐lived symptoms. Both pathways are activated in response to psychological and physical stressors and intersect at the hypothalamus.^[^ ^20^ ^]^ Though, dysregulations of the HPA axis are more associated with psychological stress and is more responsible for lingering effects from stress.^[^ ^29^ ^]^

The HPA axis consists of the hypothalamus, the pituitary gland, and the adrenal glands. Stimulation of the paraventricular nucleus of the hypothalamus by a perceived stressor results in the secretion of corticotropin releasing hormone (CRH). CRH then travels to the pituitary gland and stimulates the secretion of adrenocorticotropic hormone (ACTH), which can also act as a marker of stress dysregulation,^[^ ^45^ ^]^ into blood circulation. The binding of ACTH to cell receptors of the adrenal cortex results in the release of glucocorticoids, like cortisol, into blood circulation, in addition to other hormones implicated in psychiatric illnesses like dehydroepiandrosterone (DHEA).^[^ ^46^, ^47^ ^]^ Preclinical and clinical studies that investigated the effects of psychological stress on the brain found that the PFC, the hippocampus, and the amygdala were particularly vulnerable to chronic psychological stress.^[^ ^20^, ^25^, ^29^ ^]^ Chronic stress in rodents was found to elicit changes in glutamatergic and GABAergic signaling in the PFC and hippocampus through downregulating proteins involved in synapse formation, such as BDNF, and increasing intracellular concentrations of glutamate.^[^ ^25^, ^29^ ^]^ These observations are corroborated by findings of dendritic atrophy and consequent losses in network connectivity in the PFC and hippocampus. Moreover, it has been shown that hyperactivity (i.e., high concentrations of intracellular glutamate) in the PFC coincides with inhibition of dopaminergic neurons in the ventral tegmental area,^[^ ^26^ ^]^ which ultimately results in anhedonia, a symptom of depression. Converse to the effects observed in the PFC and hippocampus, upregulation of BDNF and increases in dendritic density were observed in the amygdala in response to chronic stress in rodents. Clinical imaging studies of patients diagnosed with MDD, BPD, PTSD, and schizophrenia found volume reductions in the PFC and hippocampus and reduced functional connectivity. However, there are conflicting accounts about the volume of the amygdala, with some reporting increases, and other reductions in amygdala volume.

The high concentration of glucocorticoid and mineralocorticoid receptors found in these regions, in addition to glucocorticoid involvement in mediating synaptic plasticity, implicates cortisol as a major contributor to the observed changes in connectivity and morphology. Cortisol is the primary glucocorticoid secreted upon activation of the stress response. Cortisol secretion naturally follows a diurnal cycle with cortisol levels being higher in the morning and lower in the evening. Cortisol has been implicated, in some way, with most neuropsychiatric illnesses. Though, for some illnesses, such as MDD and PTSD,^[^ ^48^, ^49^ ^]^ there have been conflicting studies that indicated elevated levels in some subsets, and deflated levels in others. Reflecting this implied heterogeneity of cortisol's involvement in stress‐related NPIs, there are multiple theories as to how cortisol plays a role in their pathophysiology.^[^ ^25^ ^]^ The most explored theories investigate how cortisol impacts regulation of the HPA axis at a systems level, the effects of cortisol on glutamatergic synapses in regions involved in regulating the stress response,^[^ ^25^, ^50^ ^]^ and how cortisol mediates the immune response in the peripheral and central nervous systems (discussed in the following section). Dysregulations in any of these areas can result in cognitive and behavioral changes symptomatic of some neuropsychiatric illnesses. At a systems level, cortisol acts to regulate the stress response, and therefore its own secretion, through negative feedback inhibition of the pituitary gland, the locus coeruleus,^[^ ^51^ ^]^ the hypothalamus, and the hippocampus, while increasing the activity of the amygdala and the NAc.^[^ ^20^, ^25^ ^]^ At a cellular and molecular level, the binding of cortisol to mineralocorticoid and glucocorticoid receptors in the CNS precipitates morphological and functional changes at the synaptic and network levels through genomic and nongenomic mechanisms. When cortisol binds to GRs or MRs, there are immediate changes to neuronal excitability and activity,^[^ ^52^ ^]^ followed by receptor translocation to the nucleus. At the nucleus, GRs and MRs act as transcription factors and regulate the expression of genes that participate in synaptogenesis and proper synaptic function.^[^ ^50^ ^]^ The consequences of gene expression regulation are not immediate, and can take hours or days to develop.^[^ ^53^ ^]^

The autonomic nervous system (aka the peripheral nervous system) is divided into two parts, the sympathetic nervous system (SNS) and the parasympathetic nervous system (PNS), that innervate smooth muscle tissue, glands, and cardiac muscles in the periphery. Both divisions work in parallel and inhibit the activity of the other. The sympathetic division consists of cholinergic preganglionic neurons in the spinal column and noradrenergic postganglionic neurons that control processes such as blood vessel constriction, airway relaxation, heartbeat acceleration, glucose production, pupil dilation, and salivary gland inhibition.^[^ ^51^ ^]^ Cholinergic preganglionic neurons of the PNS originate in the brain stem and innervate cholinergic postganglionic nerves that regulate most processes controlled by the SNS. Some processes, though, are only controlled by one division, such as sweat glands only being innervated by the SNS and lacrimal glands only being innervated by the PNS.

Preganglionic nerves of the SNS also innervate the adrenal medulla of the adrenal glands. Together, these SNS nerves and the adrenal medulla form the SAM axis. Activation of the SAM axis, in response to a perceived stressor, occurs via direct connections from the hypothalamus, the locus coeruleus, and the rostral ventrolateral medulla (not depicted in Figure 1) of the CNS.^[^ ^54^ ^]^ Upon activation, the adrenal medulla releases adrenaline and noradrenaline into blood circulation that essentially overstimulate the postganglionic nerves of the SNS resulting in the sympathetic stress response characterized by increased heart rate, sweat stimulation, blood pressure increase, increase in respiration rate, etc. Abnormalities in measurements of physical stress biomarkers such as heart rate variability, skin conductivity, and respiration rate, are reflective of autonomic dysfunction and are implicated as symptoms of MDD, PTSD, and anxiety disorders. In addition to catecholamines the adrenal medulla releases hormones which have been proposed as biochemical markers of autonomic dysfunction, such as neuropeptide‐y.^[^ ^55^, ^56^ ^]^

### 2.3 Connections between the Peripheral Immune System and the CNS

Correlations between peripheral cytokine levels and neuroinflammation have been well established for most neuropsychiatric illnesses, specifically, the common inflammatory markers TNF‐α, IFN‐α, IL‐1β, IL‐6, and C‐reactive protein. However, precise mechanisms of how cytokines induce neuroinflammation or how neuroinflammation can result in elevated peripheral cytokine levels is not fully understood and will depend on the illness state of the patient. For some neurological illnesses, the fundamental causes of neuroinflammation are relatively clearer. The majority of cytokines contributing to neuroinflammation, for a neurodegenerative disorder like Alzheimer's, originate from microglia, the resident immune cells in the CNS, and stromal cells reacting to homeostatic imbalances in the CNS.^[^ ^57^, ^58^ ^]^ For neuroinflammatory diseases like multiple sclerosis or meningitis, cytokine production in the CNS originates from leukocytes that have invaded the brain parenchyma due to a disrupted blood brain barrier (BBB) caused by chronic inflammation in the peripheral.^[^ ^57^ ^]^

Causes for neuroinflammation and elevated peripheral cytokine levels in psychiatric illnesses, especially those related to stress, are not as clear. However, theories, developed from murine models, are largely centered around microglia mediated neuroinflammation and BBB modulation. Activation of the SAM and HPA axes results in the release of glucocorticoids and catecholamines that bind to leukocyte receptors and subsequently stimulate leukocyte proliferation and consequent cytokine production in the peripheral.^[^ ^51^ ^]^ Peripheral cytokines can proceed to bind with receptors at the BBB that can modulate its permeability to larger and more hydrophilic molecules, enable the translocation of leukocytes from the peripheral to the CNS, or transactivate glial cells,^[^ ^51^ ^]^ specifically astrocytes and microglia, to release cytokines and reactive oxygen species into the brain parenchyma.^[^ ^59^, ^60^ ^]^ Glucocorticoids and catecholamines released after activation of the stress response, can also directly lead to cytokine production in the CNS. After crossing the BBB, these molecules can bind to their respective receptors expressed by microglia. Whether activation of microglia results in the release of anti‐ or proinflammatory cytokines is dependent on the duration of activation and the conditions under which activation occurs.^[^ ^6^, ^51^, ^61^, ^62^ ^]^ Glial cell mediated neuroinflammation plays a major role in some pathophysiological models of schizophrenia,^[^ ^6^, ^63^ ^]^ MDD,^[^ ^60^, ^61^, ^64^, ^65^ ^]^ and PTSD.^[^ ^66^, ^67^, ^68^ ^]^ One of note involves the microbiota–gut–brain axis, which is outside the scope of this review.^[^ ^69^ ^]^

### 2.4 Peripheral Biochemical Markers Implicated in Neuropsychiatric Illnesses

Biomarkers can be described as any substance, structure or indicator being biological or chemical in nature used to detect changes in an organism caused by environmental, pathophysiological or physical causality. The measured response of biomarkers takes into account molecular interactions as well as interactions at cellular, biochemical, and physiological level.^[^ ^103^, ^104^ ^]^ In neuropsychological conditions, some significant contributory stress biomarkers are BDNF, oxidative stress, salivary alpha amylase, cortisol, serotonin, and dopamine, to name a few. **Table** **1** summarizes some SBMs that have been implicated in Axis I psychological disorders. For each clinical study listed, both psychological assessment tools and a biosensor were employed to evaluate the underlying contributory physiological factors in mental health. Their inclusion is meant to show that there is clinical evidence, though most is small scale that supplements the large body of preclinical and theoretical evidence discussed in the previous section. An important feature of Table 1 is that many of the SBMs are linked to multiple NPIs.

**Table 1:** Biomarkers of psychological disorders, where the biomarkers have been analyzed using biotechnology and corroborated with psychological diagnostic tools.

Psychological disorder	Contributory biomarker	Biofluid	Assay used	Psychological assessment	Sample size	Refs.
Depression	Brain‐derived neurotrophic factor (BDNF)	Blood serum	Immunoassay	Inventory of depressive symptoms and CIDI	*N* = 1751 Incident depression (*n* = 153), In remission (*n* = 420) Persistent depression (*n* = 310), Nondepressed controls (*n* = 868)	[70]
		Blood serum and lymphocytes	Sandwich ELISA assays	HRSD‐24	*N* = 215 Depression (*n* = 90) Bipolar disorder (*n* = 15) History of suicide attempt (*n* = 14) Healthy control (*n* = 96)	[71]
		Blood plasma	Immunoassay	MADRS	Major depressive disorder (MDD) patients, Remission group *n* = 38 Nonresponder group *n* = 10	[72]
Depression	BDNF	Blood serum	ELISA	PHQ‐9	Patients with acne vulgaris, and depression (*n* = 20) and without depression (*n* = 98) Healthy control (*n* = 59)	[73]
	Ferritin	Blood serum	Serum ferritin: immuno‐turbidimetric assay Body iron: calculated using a specific formula	CES‐D	Young adults (17–25 years) Women *n* = 562 and Men *n* = 323	[74]
		Blood plasma	Immunoassay	CES‐D	Obese group *n* = 28 Normal group *n* = 27	[75]
	Salivary alpha amylase	Saliva	Salivettes technology	BDI‐II	*n* = 30 Depressed *n* = 15 Nondepressed *n* = 15	[76]
		Saliva	Dipstick‐based assay	HARS and HDRS	*N* = 90 *n* = 30 anxious *n* = 30 depressed *n* = 30 control	[77]
	Oxidative stress and cytokines	Blood serum	ELISA	SCID‐5	Depressed patients *N* = 247 Healthy control *N* = 248	[78]
		Blood plasma	Chromatography for oxidative stress and enzyme‐linked immunosorbent assay for inflammatory cytokines	SCID, HDRS, and IDS	Major depressive disorder (MDD) *n* = 20 Matched healthy control *n* = 20	[79]
		Blood serum	Superoxide anion (O‐2) measured by cytochrome c reduction assay; cytokines measured using ELISA	HAM‐D	Depressed group *n* = 29 Healthy control *n* = 30	[80]
Bipolar affective disorder (BD)	Brain‐derived neurotrophic factor (BDNF)	Blood serum	Sandwich ELISA technology	SCAN 2.1, YMRS	Bipolar mania group *N* = 21 Healthy control *N* = 19	[81]
		Blood serum	ELISA	SCID, YMRS, HDRS	Bipolar patients *N* = 83 with (bipolar mania *n* = 61, bipolar depressive *n* = 22) Healthy control *N* = 222	[82]
	Cytokines	Blood plasma	Immunoassay	SADS‐L, YMRS, and HDRS	Bipolar I *N* = 234 Bipolar II *N* = 260 Healthy control *N* = 140	[83]
Bipolar affective disorder	Cytokines	Whole blood	ELISA	BPRS, YMRS	Bipolar mania *N* = 37 Normal control *N* = 74	[84]
	Oxidative stress	Blood serum	Spectrophotometry	SCID‐I, YMRS	BD patients with first‐episode mania *N* = 30 BD patients with more than one manic episode *N* = 52 Healthy control *N* = 45	[85]
		Blood plasma	Spectrophotometry	SCID, HAM‐D, YMRS	Bipolar patients (BD) *N* = 29 Age matched healthy control *N* = 28	[86]
PTSD	Cortisol and DHEA	Saliva	ELISA	BDI, Echeburua's severity of symptom scale of post‐traumatic stress disorder	Female victims of intimate partner violence diagnosed with depression *N* = 36, with PTSD *N* = 18 Healthy control who are nonabused *N* = 31	[87]
PTSD	Cortisol and DHEA	Saliva	Immunoassay and Dynex Spectrophotometer	PSS‐SR, CES‐D	Diagnosed with PTSD *n* = 15 Comorbid with depression and PTSD *n* = 18 No pathology (healthy) group *n* = 27	[88]
		Hair	Liquid chromatography tandem mass spectrometry (LC‐MS/MS)	UCLA PTSD Reaction Index for DSM IV Child Version, STAI, CES‐D, SoC, and SoFC scale	Female adolescents from the West Bank affected by the Israeli–Palestinian conflict *N* = 92 divided into: Nontrauma group *n* = 36 Trauma exposed *n* = 17 PTSD subgroup *n* = 39	[89]
Anxiety disorders	Pregnenolone sulfate in generalized anxiety disorder (GAD)	Blood plasma	Chromatography	M.I.N.I., CGI	Men with depression *n* = 20 Men with anxiety *n* = 20 Controls = 30	[90]
		Blood plasma	High‐performance liquid chromatography (HPLC)	HARS	Males diagnosed with GAD *N* = 8 Healthy control *N* = 8	[91]
	γ‐Aminobutyric acid (GABA) in panic disorder	_ Lower occipital cortex analyzed	Spectroscopy using a 2.1‐T, 1‐m bore magnet with a spectrometer, and actively shielded magnetic field gradients	CRAS, PDSS, HAM‐A, and HAM‐D	Patients with panic disorder *N* = 14 Healthy control group *N* = 30	[92]
Anxiety disorders	γ‐Aminobutyric acid (GABA) in panic disorder	Lumbar Cerebrospinal fluid (CSF)	High‐performance liquid chromatography (HPLC)	HAM‐A, MADRS	Subjects with panic disorder *n* = 11 Control group *n* = 6	[93]
Schizophrenia	BDNF	Blood serum	ELISA	BPRS	Antipsychotic‐naive (*n* = 15) Medicated patients (*n* = 25) and normal controls (*n* = 40)	[94]
		Blood serum and blood plasma	ELISA	BSIP, BPRS, Neuro‐psychological Tests: CVLT, TAP, ToH, CPT, MWT‐A, CPS‐3	Male patients only At risk of mental state (ARMS) *n* = 16 First episode psychosis (FEP) *n* = 6 Chronic schizophrenia (CS) *n* = 11	[95]
		Blood plasma	ELISA	PANSS, GAF	Chronic schizophrenia (CS) patients *N* = 68 Healthy subjects *N* = 32	[96]
	Cortisol, dehydroepiandrosterone sulfate (DHEAS), and DHEA	Blood serum	Immunoassay	PANSS, STAI	Schizophrenia patients *N* = 43 Healthy control *N* = 18	[97]
		Blood serum for cortisol and blood plasma for DHEAS	Immunoassay	BPRS, SANS, HAMD, HAMA, BPRS‐Pos, PSS, and SCID‐NP	First episode psychosis (FEP) *N* = 23 (follow‐up) Healthy control *N* = 15 (follow‐up)	[98]
		Blood serum	Radioimmunoassay	PANSS, HAM‐D, HAM‐A, CGI, LHA	First episode of psychosis (patient group) *N* = 37 Healthy control *N* = 27	[99]
		Blood plasma	Immunoassay	SCID‐P, PANSS	Patients diagnosed with schizophrenia *N* = 23 Healthy control *N* = 23	[100]
	Cytokines	Blood serum	ELISA	SCID, PANSS	Cases (schizophrenia patients) *n* = 26 Healthy control group *n* = 26	[101]
		Blood serum	ELISA	CGI severity scale, PANSS	Schizophrenia patients (cases) *N* = 41 Healthy control *N* = 25	[102]

On drawing attention to BDNF, which spreads widely in the central and autonomic nervous systems, it is found to play an essential role in survival of neurons in addition to their differentiation during brain development, neuroplasticity, and synaptic transmission. Moreover, BDNF is involved in long‐term potentiation in the hippocampus for learning and memory.^[^ ^105^ ^]^ BDNF is considered to be a potential diagnostic biomarker for both mood and anxiety disorders, schizophrenia, epilepsy, and even motor movement disorders like Alzheimer's and Parkinson's dementia.^[^ ^34^, ^106^, ^107^ ^]^ Since psychological disorders are multifaceted in etiology and heterogenous in symptom expression, it is practically not possible to dote one contributory biomarker for a specific illness. For instance, decreased BDNF has been linked to cognitive symptoms of schizophrenia where research exhibits that on providing cognitive remediation there is a significant increase in BDNF levels and this cocontributes in improving global cognition, learning, verbal memory, and processing speed in schizophrenic clients.^[^ ^106^, ^108^ ^]^ Collaterally, a study by Kropp et al.^[^ ^109^ ^]^ has shown that typical antipsychotic drugs used to treat schizophrenia tend to elevate oxidative stress by altering levels of antioxidant enzyme causing lipid peroxidation in them. The imbalance between prooxidants and antioxidants impairs the glutathione levels, decreases the activities of antioxidant defense systems, and elevates malondialdehyde as schizophrenia progresses.^[^ ^110^, ^111^ ^]^ It can thus be said that both BDNF and increased oxidative stress (OS) contribute to schizophrenia.^[^ ^111^ ^]^ OS is not just limited to a specific disorder, its harmful effects have been exemplified in a plethora of chronic diseases like cancer, cardiovascular diseases, respiratory diseases like asthma and chronic obstructive pulmonary disease, rheumatoid arthritis and renal failure;^[^ ^112^ ^]^ as well as in neurogenerative disorders like Alzheimer's, Huntington's, and Parkinson's disease, traumatic brain injury, amyotrophic lateral sclerosis;^[^ ^113^ ^]^ and other neuropsychological conditions like bipolar affective disorder,^[^ ^114^ ^]^ and autism spectrum disorder.^[^ ^115^ ^]^

For complex mood disorder like bipolar affective where like a sine wave the mood fluctuates between depression and mania patched with euthymia, BDNF and OS predominate as peripheral biomarkers again with an increased circulation of proinflammatory cytokines as this condition progresses.^[^ ^116^ ^]^ While BDNF is found to decrease in bipolar, the thiobarbituric acid reactive substances levels generated during oxidative stress increase along with an elevation of levels of enzymes nitric oxide (NO) and superoxide dismutase supporting lipid peroxidation leading to bipolar affective disorder.^[^ ^117^ ^]^ Certain potential neuroimaging biomarkers have also been identified using neuroimaging techniques in this condition. Using structural neuroimaging, T2‐weighted magnetic resonance images (MRIs) have revealed an increase in white matter hyperintensities (WMH), making it a potential endophenotype of bipolar disorder and moreover WMH is not found to be present in unipolar depression except for the geriatric populace suffering from major depressive disorder.^[^ ^116^, ^118^, ^119^ ^]^ A meta‐analysis of studies on bipolar in children and adolescents using MRI morphometry to measure amygdala volume on at least three resonance slices have shown significantly smaller amygdala volumes in children and adolescents with bipolar compared to their control with a standardized mean difference −0.74 and 95% confidence interval −1.36 to −0.15.^[^ ^120^ ^]^ Lastly, with technological advancements in whole genome sequencing and genome‐wide association studies, an association has been reported with BDNF, catechol‐O‐methyltransferase, serotonin transporter (5‐HTT) genes, and bipolar disorder on comparing with control group; however, the context of specificity still remains clouded as these associations are found in other psychiatric conditions as well like schizophrenia, major depression, and eating disorder.^[^ ^116^, ^121^ ^]^ Other polymorphisms that serve as strongest risk factors in bipolar are calcium voltage‐gated channel alpha 1C subunit (*CACNA1C*) and ankyrin 3 (*ANK3*).^[^ ^122^, ^123^ ^]^

Research by Fullana et al.^[^ ^124^ ^]^ has unraveled that obsessive compulsive disorder has more inviolable contributory neurocognitive biomarkers (like poor cognitive flexibility, attention and planning, lack of inhibition, impaired verbal and nonverbal memory, reduced visuospatial ability) than biochemical, neurophysiological, and neuroimaging biomarkers. Even though increased levels of cortisol and antibasal ganglia antibodies positivity is found to be present along with fractional anisotropy revealing increased anterior limb of the internal capsule and a reduction in the fractional anisotropy of the genu of the corpus callosum, they have weak associations. However, it is not the same for the entire spectrum of anxiety disorders. Tocchetto et al.^[^ ^125^ ^]^ documented the role of polymorphism Val66Met (a Met allele of the BDNF) in anxiety disorders of children and adolescents where DNA was extracted using salivary samples. Four metabolomic biomarkers, namely, *N*‐methylnicotinamide, aminomalonic acid, azelaic acid, and hippuric acid involved in three metabolic pathways: tryptophan–nicotinic acid metabolism, lipid metabolism, and tyrosine–phenylalanine pathways are also found to be contributory factors of underlying distress and anxiety disorders.^[^ ^126^ ^]^

Biomarker research on ADHD in children has linked dopamine transporter gene (DAT1) polymorphism with lowered response inhibition and also enhanced the pharmacotherapeutic efficacy of methylphenidate.^[^ ^127^ ^]^ A meta‐analysis of studies from 1969 to 2011 on ADHD done by has shown significant contribution of norepinephrine (NE), 3‐methoxy‐4‐hydroxyphenylethylene glycol (MHPG), monoamine oxidase (MAO), zinc (Zn), and cortisol in ADHD medications and also supported lead and ferritin to be meaningful biomarkers managing cognitive functioning.^[^ ^128^ ^]^ Attention deficit is no more considered to be a behavioral problem but a neurodevelopmental condition presently for such scientific research advancements.

### 2.5 Wearable Biosensors

**Figure 2:**
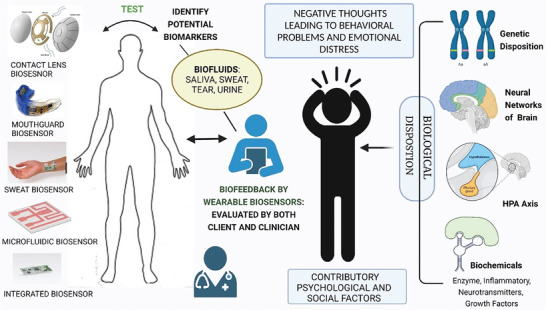
Applicable integration of biosensors in psychiatric diagnosis and treatment. Created using Biorender.com.

What sets wearable sensors apart from POC or laboratory‐based detection methods is their ability to provide continuous SBM monitoring without supervision and outside the clinic, i.e., without the need for appointments or hospitalization. In this review, we consider point‐like measurements as measurements taken regardless of the natural circadian and ultradian rhythms of SBMs. We therefore consider continuous monitoring as measurements taken frequently and periodically so as to capture these variations and discriminate them from real signals. The potential benefit of this approach is particularly significant for clients suffering from neuropsychiatric illnesses, which are characterized by psychological stressors and exogenous factors that cause symptom presentation and prevent remission. From the perspective of the patient, wearable sensors enable self‐monitoring of symptoms so that they may better understand how their behaviors and their environment affect thems. From a clinician's perspective, wearable sensors are tools that can help with diagnostics and guiding treatment plans through the objective measure of biomarkers (**Figure** **2**). They can provide immediate biofeedback to a patient and their clinician about the physiological state of a patient in the context of their daily life to the patient and their clinician.

Currently, the field of wearable biosensors for detecting and monitoring psychiatric and neurological biochemical markers is still in its infancy. The vast majority of studies focus on the electrochemical, noninvasive detection of cortisol in sweat for potential applications like monitoring disruptions in circadian rhythm or for general stress management.^[^ ^15^, ^17^ ^]^ Cortisol, as a SBM, is believed to be more specific to psychological stress than autonomic stress markers like skin conductivity, heart rate variability, or body temperature. However, as was discussed throughout the previous sections, cortisol does not correlate with one specific NPI. For a WBS that targets cortisol to be an effective ancillary tool in clinical practice, other SBMs must be simultaneously targeted. Additionally, the time‐dependent nature of NPIs warrants WBSs capable of long‐term continuous monitoring, which, here, refers to measurements that are taken frequently and periodically so as to differentiate natural fluctuations from real signals. Both general performance requirements are dependent on the choice of biofluid for sampling. Not all SBMs can be reliably found in every peripheral biofluid and their concentrations become more dilute as they partition from the blood to externally secreted biofluids. Adding onto the complexity is that the other SBMs mentioned throughout this review are also not unique to a specific NPI, and that with increasing SBM size, there is an increase in chemical complexity and a decrease in concentration.

The following sections are meant to inform the reader of alternative biofluids to sweat, relevant WBSs demonstrated thus far for sampling these biofluids, and the current state of wearable sweat sensors for targeting SBMs. These topics will be discussed in the context of the previously described design considerations with an emphasis on multiplexation. Overall, the intention of this section is to showcase the technological advancements made toward the on‐body detection of SBMs with WBSs, while also highlighting overlooked and prospective areas of research.

### 2.6 Interstitial Fluid

#### 2.6.1 Overview of Wearable Biosensors for Sampling Interstitial Fluid

Interstitial fluid is the fluid that bathes the extracellular space in tissues. To put simply, endothelial cells separate the blood from interstitial fluid and act as a semipermeable membrane that allows the exchange of ions, lipophilic molecules, like cortisol and DHEA, and low‐molecular weight hydrophilic molecules between the blood and ISF.^[^ ^129^, ^130^ ^]^ For macromolecules, their large sizes result in their significant dilution when being transported from the blood to ISF. Subsequent partitioning of biomolecules between the ISF and secretory glands further dilutes their concentration in secreted biofluids with some of the larger biomarkers having concentrations in the picomolar and femtomolar ranges.^[^ ^7^ ^]^ Therefore, real‐time, sensitive detection of NPBs in sweat, tears, and saliva with a wearable biosensor becomes more difficult. Thus, the higher biomolecule concentrations in the ISF relative to the other peripheral biofluids have the clear benefit of aiding the sensitive detection of target analytes. However, ISF is not naturally secreted like saliva, tears, and sweat. Access to the biofluid with a wearable device requires either forced extraction using a noninvasive method like reverse‐iontophoresis or direct sampling using transdermal needles.^[^ ^131^, ^132^, ^133^, ^134^, ^135^ ^]^

Reverse iontophoresis, in this context, requires an electric current to be imposed across the skin such that the flow of electrons moves from the cathode (i.e., where molecules are reduced) to the anode (i.e., where molecules are oxidized). Through the penetration of the electric field into the subdermal layer, ions in the fluids within the layer diffuse to their respective electrodes. A positive ion diffuses to the cathode while a negative ion diffuses to the anode in this case. In addition, an osmotic pressure is generated which causes ISF to flow toward the cathode.^[^ ^136^ ^]^ Within this ISF are neutrally charged molecules and positively charged ions that selectively passed through the skin. The skin's intrinsic negative charge at physiological pH hinders the transdermal flux of anions. Although iontophoresis has been demonstrated to be effective at enhancing the transdermal flux of species of varying size and charge,^[^ ^137^ ^]^ and reverse‐iontophoresis has been shown to be effective in transdermal extraction,^[^ ^138^ ^]^ there are challenges. An often cited issue is that iontophoresis has been known to cause minor reactions like itching, skin irritation, or erythema at the site of administration, and, in rare, severe cases, burning or scarring with prolonged usage of the device.^[^ ^139^ ^]^ Aside from this drawback, perhaps the biggest challenge opposing the use of reverse‐iontophoresis for ISF extraction is the high fluidic resistance imparted by the dermal extracellular matrix, which severely limits the amount of dermal ISF that can be extracted at a given time.^[^ ^129^ ^]^ Using iontophoretic delivery of hyaluronic acid,^[^ ^131^ ^]^ microneedles,^[^ ^140^ ^]^ or heat in combination with reverse‐iontophoresis to improve the ISF extraction rate have proven effective for the continuous monitoring of glucose.^[^ ^141^ ^]^ However, whether these systems can be translated for other biochemical markers is unclear.

A minimally invasive alternative to iontophoretic extraction of ISF is to use microneedle arrays to directly sample the ISF. Microneedles are micrometer‐sized, sharp protrusions that,^[^ ^142^ ^]^ unlike the invasive needles used in commercially available continuous glucose monitors,^[^ ^143^ ^]^ are designed to pierce the outermost layer of skin (i.e., the stratum corneum) to access ISF in the epidermis rather than ISF in subcutaneous tissue. Consequently, the microneedles do not pierce the capillary bed. Fabrication of the biosensor on the tips of the microneedles (i.e., on‐tip sensing) means that analytes in ISF can be directly sampled in real‐time without extraction of the fluid.^[^ ^142^ ^]^ This is ideal for continuous monitoring. The natural flux of biomolecules into and out of the ISF means that fresh sample is periodically delivered to the active layer without the need of an external pressure gradient and subsequent flow control. Additionally, the adjacency of ISF to the blood means the lag‐time between biomarker generation and detection should be lower compared to detection with devices that sample outside the body.^[^ ^130^ ^]^ So far, studies demonstrating wearable devices using microneedles have shown they can detect biomarkers like glucose,^[^ ^144^, ^145^, ^146^, ^147^, ^148^, ^149^, ^150^ ^]^ lactate,^[^ ^151^, ^152^ ^]^ ions,^[^ ^153^, ^154^, ^155^ ^]^ and drugs,^[^ ^156^, ^157^ ^]^ with on‐tip sensing.

An obstacle for in situ sensing with microneedles is they must manage sensitive analyte detection in a complex biological environment. At the nanoscopic and molecular levels, the heterogeneous composition, higher molar concentrations of molecules and ions, presence of degrading species, and the surrounding extracellular matrix all increase the possibility of nontarget interference, biofouling of the sensor, and degradation of the biorecognition elements. Anti‐biofouling membranes like Nafion, cellulose acetate, or polyethylene glycol, like how they are used in continuous glucose monitors,^[^ ^135^, ^158^, ^159^ ^]^ are frequently used in microneedle technology for improving sensor longevity and selectivity, however, they have mainly been demonstrated for smaller biomarkers detected enzymatically. There are examples of microneedle platforms, demonstrated with murine models, for the detection of biomacromolecules that show the technology's promise for detecting more complex biomolecules.^[^ ^160^, ^161^, ^162^, ^163^, ^164^, ^165^ ^]^ However, in such studies, the devices were demonstrated on ex vivo or in vivo murine models with only one demonstrating the long‐term stability of the sensor while in vivo.^[^ ^166^ ^]^ As such, selective and sensitive detection of more complex biochemical markers with microneedles may be challenging for applications that require long‐term continuous monitoring.

Microneedle platforms are relatively easy to adapt for multiplex applications because multiple, separate arrays can be manufactured and differentially functionalized on a single substrate. So far, multiplex, in situ microneedle platforms have been demonstrated for the detection of electrolytes and metabolites.^[^ ^152^, ^157^, ^165^, ^167^, ^168^, ^169^ ^]^ However, most of these platforms were not demonstrated on‐body. An exception is the robust, multiplex platform presented by Tehrani et al. for the multiplex, enzymatic detection of glucose and lactate or glucose and ethanol.^[^ ^168^ ^]^ The disposable biosensor consisted of micromachined poly(methyl methacrylate) microneedles separated into two distinct working electrode arrays. Each working electrode was comprised of a layer of sputtered conducting metals, a layer of poly*‐o*‐phenylenediamine for anti‐biofouling, a layer of the appropriate oxidase enzyme mixed with chitosan, and a final layer of PVC containing a nonionic surfactant as the diffusion limiting layer. The group demonstrated that the biosensor was capable of continuously monitoring the change in glucose and ethanol over a 5 h period following consumption of wine and a meal. Likewise, the same was demonstrated for glucose and lactate following high‐intensity exercise and meal consumption.

Microneedles, while promising for minimally invasive diagnostics and monitoring, face several intrinsic limitations. Their operation requires skin penetration, which can result in irritation, blistering, or even immune responses due to the introduction of foreign materials. Biofouling may also pose risks of adverse side effects. Material selection is critical, as unsuitable materials could interfere with detection accuracy in bodily fluids. Effective microneedle design must balance penetration depth to ensure contact with interstitial fluid without causing excessive pain or inflammation. Parameters such as tip diameter, length, thickness, and angle significantly influence insertion ease, patient comfort, and diagnostic reliability. For instance, a study by Römgens et al. highlighted the importance of tips with diameters below 15 µm for optimal penetration,^[^ ^170^ ^]^ while Gill et al. found pain increased proportionally to microneedle length.^[^ ^171^ ^]^ However, even with standardized dimensions, only a subset of microneedles in an array might achieve proper dermal insertion, underscoring the challenges of ensuring consistent performance.^[^ ^129^ ^]^

#### 2.6.2 Application of ISF‐Sampling Wearables: Therapeutic Drug Monitoring

Discerning effective doses, determining which medication(s) are best for the client, and, in some cases, if medication is necessary, can be challenging considering the complexity of neuropsychiatric illnesses and how they are diagnosed. Complicating these issues are the unavoidable variabilities between each individual patient and drug‐specific challenges such as: narrow therapeutic windows, like with apomorphine or levodopa in the treatment of Parkinson's disease; adverse side effects, potential ineffectiveness, withdrawal symptoms when trying to change medications, and long waiting times before drugs take effect, as are witnessed with common antidepressant and antianxiety medications.^[^ ^156^, ^172^, ^173^, ^174^, ^175^, ^176^ ^]^ Unfortunately, these pharmaceutical complications are at the detriment of the patient who is expected to manage their lives despite these biochemical disruptions. The multiplex detection of biomarkers to gauge a client's response to certain medications would no doubt be beneficial.

**Figure 3:**
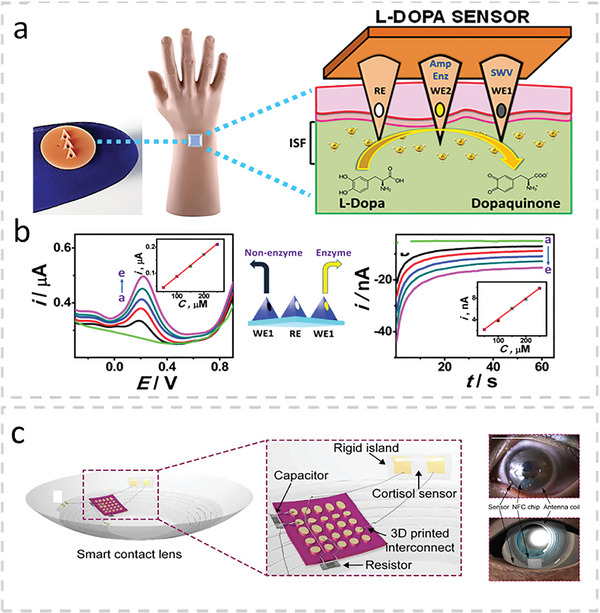
a) Microneedle‐based wearable sensor for the detection of L‐Dopa in ISF. Adapted with permission.^[^ ^156^ ^]^ Copyright 2019, American Chemical Society. b) A schematic with data showing the dual mode sensing capabilities of the microneedle sensor. c) Wearable contact lens for the detection of cortisol in tears. Adapted with permission.^[^ ^179^ ^]^ Copyright 2020, American Association for the Advancement of Science.

Therapeutic drug monitoring is, thus far, the only proposed neuropsychiatric application of wearable, ISF‐sampling biosensors and all examples are of monitoring drugs for treating Parkinson's disease.^[^ ^156^, ^172^, ^177^, ^178^ ^]^ The sensor developed by Goud et al.^[^ ^156^ ^]^ used microneedles for the detection of levodopa in ISF (**Figure** **3**a). The sensor used two microneedles at which one enzymatically detected levodopa using chronoamperometry and the other nonenzymatically detected levodopa using square wave voltammetry (Figure 3b). Both microneedles were determined to operate within the range of 0.25–3.0 µm with an LOD of 0.25 µm in artificial ISF. The dual‐mode sensor proved advantageous when monitoring L‐Dopa in the presence of the inhibitory drug and L‐Dopa analogue, C‐Dopa. Additions of C‐Dopa to artificial ISF with an L‐Dopa concentration of 80 µm resulted in signals at both electrodes that were discernable from the L‐Dopa signal. The group also demonstrated that the sensor could detect L‐Dopa in ISF in an ex vivo mouse‐skin model and tissue‐mimicking phantom gel. Lastly, the group demonstrated the device capable of continuous real‐time monitoring of L‐Dopa in artificial ISF over a period of 110 min while in the presence of biofouling proteins and other interfering agents.

### 2.7 Saliva

Saliva does not require stimulation to receive ample volumes of the fluid, and it has abundant quantities of certain neuropsychiatric biomarkers like BDNF and α‐amylase, which make saliva an excellent biofluid for laboratory‐based and POC detection. For wearable sensors, saliva presents challenges for applications that require continuous monitoring. The secretion rate and composition of saliva, which itself is dependent on secretion rate, are dependent on a variety of factors like gland type, time of day, type of stimulation, age, and medication.^[^ ^180^ ^]^ These factors make continuous collection of saliva with a wearable device over long time periods difficult, despite the total secretion rates for stimulated and passively secreted saliva being about the same.^[^ ^130^ ^]^

The device form should also be considered. Most of the wearable sensors developed for measuring biomarkers in saliva are in the form of mouthguards.^[^ ^181^, ^182^, ^183^ ^]^ The need for the device to be in the mouth means it will be subject to a variety of contaminants throughout day. Consequently, a wearable platform for the continuous monitoring of SBMs in saliva should be used for biomarkers whose variability throughout the day is negligible or can be mitigated by careful choice of sampling rate. So far, no wearable platforms that target SBMs in saliva have been reported.

### 2.8 Tears

Tears are the least explored of the peripheral biofluids. Their underutilization is understandable considering that for years the fitness monitoring and diabetes‐management markets were the primary driving forces behind wearable sensing technology,^[^ ^184^ ^]^ and markers like glucose and lactate can be more easily obtained from sources other than tears. Additionally, tears as a biofluid for wearable sensing suffer from similar problems as saliva. Like with saliva, the secretion rate and composition vary with tear type. Another point of consideration is that sample volumes will be low because the tear film (i.e., conjunctival tears) that constantly covers the eye surface will be the primary sampling fluid and constant stimulation of tears would be impractical for everyday applications.^[^ ^185^ ^]^ Their close association with the central nervous system, however, gives them access to NPBs, such as those associated with Alzheimer's or those associated with Parkinson's that are not found in other biofluids.^[^ ^186^, ^187^ ^]^ Despite this advantage, there are only a handful of examples of wearable tear sensors as mentioned above, only one of which was used for the detection of an SBM.^[^ ^179^ ^]^ In the study, the group demonstrated the sensing performance and body‐compatibility of a wireless immunosensor integrated into a soft contact lens for the detection of cortisol in human tears (Figure 3b). Their sensor could detect cortisol within its physiological range in tears (1–40 ng mL^−1^) and was shown to cause no damage to the eye after 12 h of being worn. Whether this sensor could continuously measure cortisol levels in the tear film over that 12 h period was not demonstrated. This piece of information, in addition to a comparison of the results with a standard, is needed to further validate the device's potential.

### 2.9 Sweat

Eccrine sweat is currently the most investigated biofluid for the detection of NPBs with wearable biosensors. Though biomarker concentrations are more dilute compared to ISF, it has several advantages that make it more attractive for continuous monitoring of biomarkers with wearable devices. Eccrine sweat glands make up the majority of sweat glands on the body—with most regions containing ≈200 eccrine sweat glands cm^−2^.^[^ ^188^ ^]^ Contrasting with the glands of saliva and tears, the eccrine sweat glands are largely, but not entirely, separate from apocrine sweat glands, which prevents cross‐contamination of their secretions. Significant quantities of secreted eccrine sweat can be easily collected using noninvasive methods that rely on stimulation of the sweat glands using physical activity, heat, or a chemical stimulant like pilocarpine or carbachol delivered to the glands using iontophoresis.^[^ ^189^, ^190^, ^191^ ^]^ Secreted sweat can produce a remarkable amount of pressure per gland that facilitates sweat flow into the inlet of a microfluidic device or absorption by a wicking material.^[^ ^192^ ^]^ A large, variable collection of wearable sweat sensors have been proposed for the electrochemical,^[^ ^193^, ^194^, ^195^, ^196^ ^]^ colorimetric,^[^ ^197^, ^198^, ^199^, ^200^ ^]^ plasmonic,^[^ ^201^ ^]^ or fluorometric detection of metabolites, electrolytes, drugs, etc.^[^ ^202^ ^]^ A majority of WBS designed for the detection SBMs are tabulated in **Table** **2** along with important parameters that will be discussed throughout the rest of this section.

**Table 2:** A list of wearable biochemical sensors that have been demonstrated for the on‐body detection of neuropsychiatric‐relevant biomarkers in sweat.

Detected biomarker	Transduction element	Biorecognition element	Detection method	Sampling element	LOD	Storage lifetime/ longest on‐body, continuous measurement	Tested range	Refs.
Cortisol	Ti_3_C_2_T*_x_* MXene flakes deposited onto laser burned graphene	Antibodies	Electrochemical	Microfluidic device	3.8 pm	–	0.1–100 nm	[203]
Cortisol	Patch with integrated, three‐electrode immunosensor	Antibodies	Electrochemical	Direct contact	7.47 nm	–	100–500 nm	[204]
Cortisol	3D‐nanostructured gold electrode	Antibodies	Electrochemical	Microfluidics	1 pg mL^−1^	–	1 pg mL^−1^–1 µg mL^−1^	[205]
Cortisol	Cellulose nanocrystals– carbon nanotubes composite	MIP	Electrochemical	Perforated medical tape	2.0 ± 0.4 ng mL^−1^	–	10–66 ng mL^−1^	[206]
Cortisol	Fe_2_O_3_ deposited onto conductive carbon yarn	Antibodies	Electrochemical	Carbon yarn	0.005 fg mL^−1^	30 days, @4 °C**/**1 h^a)^	1 fg mL^−1^–1 µL	[207]
Cortisol	Graphene	Antibodies	Electrochemical	Microfluidics	0.08 ng mL^−1^	35 days, @4 °C**/**30 s^a)^	0.43–50.2 ng mL^−1^	[208]
Cortisol	PEDOT:PSS OECT	MIP	Electrochemical	Microfluidic device	2.68 µA dec^−1^	–	0.01–10 µm	[10]
Cortisol, sweat pH, skin temperature	Field‐effect transistor with In_2_O_3_ channel	Aptamers	Electrochemical	Microfluidic device	–	**/**1 min	10 fm–100 µm	[11]
Cortisol	OECT with polyanaline channel	Aptamers	Electrochemical	Nanofibers	10 pm	**/**800 s	1 pm–10 µm	[209]
Cortisol	ZnO on polyamide substrate	Aptamers	Electrochemical	Polyamide wick	30 pg mL^−1^	**/**8 h	1–256 ng mL^−1^	[210, 211, 212]
Cortisol, DHEA^b)^	Au electrodes	Antibodies	Electrochemical	Polyamide wick	0.1 ng mL^−1^,	**/**4 h	0.1 mL^−1^–200 ng mL^−1^,	[14]
					0.1 ng mL^−1^		0.1–200 ng mL^−1^	
Cortisol, glucose	Au electrodes	Antibodies	Electrochemical	Polyamide wick	–	**/**8.5 h	1–150 ng mL^−1^	[213]
Cortisol	Prussian Blue	MIP	Electrochemical	Microfluidic channels	0.2 nm	**/**60 s	10–1000 nm	[214]
Cortisol	Ni–Co metal organic framework on top of CNT electrodes	Aptamers	Electrochemical	Absorbent cloth	0.032 ng mL^−1^	7 days, @4 °C	0.1–100 ng mL^−1^	[215]
Cortisol	PEDOT:PSS OECT	Antibodies	Electrochemical	Microfluidics	2.68 µA dec^−1^	**/**20 min	10–10 000 nm	[216]
Cortisol	Carbon black/Prussian Blue nanoparticles	Antibodies	Electrochemical	Paper‐based microfluidics	3 ng mL^−1^	**/**10 min	10–140 ng mL^−1^	[217]
Cortisol	Zn‐based MOF/Prussian Blue/PEDOT:PSS	Antibodies	Electrochemical	Direct contact	0.26 pg mL^−1^	9 days, @4 °C**/**10 h	1 pg mL^−1^–1 µg mL^−1^	[218]
Cortisol	PbNPs@MIP/AuNPs	MIP	Electrochemical	Carbon nanofibers	0.35 nm	20 days, @4 °C	1–1000 nm	[219]
Cortisol, Mg^2+^, pH	Analyte sensitive membranes deposited on Ti_3_C_2_T*_x_* MXene/MWCNTs/Ag NPs composites	Polypyrrole MIP, Mg^2+^ ion selective membrane, polyaniline	Electrochemical	Microfluidic device	0.072 nm	20 days**/**≈5.5 h	1 nm–10 µm, 1 µm–10 mm, pH 3–7.5	[220]
Cortisol	Indium–titanium oxide	Aptamers	Electrochemical	Microfluidic device	8 nm	7 days, @40 °C	10 nm–5 mm	[221]
Cortisol	Graphene field‐effect transistor	Antibodies	Electrochemical	Direct contact	1.84 ng mL^−1^/1% change in resistance	8 days, RT and 36.5 °C	1–40 ng mL^−1^	[179]
Glucose, uric acid, lactate, Na^+^, NH_4_^+^, K^+^ ^c)^	Prussian Blue‐NiHCF zeolyte@AuNPs@carbon electrodes, carbon electrodes	Enzyme‐catalyzed oxidation, ionophores	Electrochemical	Iontophoresis and microfluidic device	33.65 nA µm ^−1^, 185.56 nA µm ^−1^, 6.36 nA µm ^−1^, 58.9 mV dec^−1^, 60.6 mV dec^−1^, 61.2 mV dec^−1^	100 h, 24 h	25–100 µm, 5–20 mm, 5–100 mm, 10 ‐160 mm, 2– 32 mm, 25–100 µm	[13]
Cortisol	Gold nanoparticles conjugated to PDMS	Aptamers	Surface plasmon resonance	Direct contact	0.1 nm	–	0.1–1000 nm	[222]
Cortisol	Photonic crystal hydrogel	MIP and antibodies	Change in reflectance/color	Hydrogel	1 nm for both	**/**24 h	1–10 000 nm	[19]
Cortisol, glucose, ascorbic acid	AuNPs, enzyme‐catalyzed reduction of fluorescent probes	Primary and secondary antibodies, enzymes	Colorimetric, fluorescence	Paper lateral flow assay, microfluidic device	5 ng mL^−1^, 0.1 µm, 2 µm	**/**2.5 h	5–100 ng mL^−1^, 0.1–2 µm, 5–100 µm	[223]
Cortisol	Au electrodes	Methylene blue‐functionalized aptamer	Electrochemical	Microfluidic device	0.2 pm	**/**90 min	1 pm–1 µm	[224]
Dopamine	Fiber‐based organic electrochemical transistors with polypyrrole channel	Dopamine oxidation to *o*‐dopamine quinone	Electrochemical	Polymer fiber	47.28 NCR per decade	–	1 nm–1 µm	[225]
IFN‐γ, IL‐6, IL‐8, IL‐10^d)^	ZnO on Ag electrode	Antibodies	Electrochemical	Polyamide wick	0.2 pg mL^−1^	1 h	0.2–200 pg mL^−1^ for all analytes	[226]
IL‐1β, CRP	ZnO on Ag electrode	Antibodies	Electrochemical	Polyamide wick	0.2 pg mL^−1^, 1 pg mL^−1^	30 days, @4 °C**/**6.5 h	0.2–200 pg mL^−1^, 1 pg mL^−1^–10 ng mL^−1^	[227]
TNF‐α	Graphene field effect transistor	Aptamers	Electrochemical	Janus membrane	0.31 pm	15 days	0.5–500 pm	[228]
TNF‐α	ZnO on Ag	Antibodies	Electrochemical	Polyamide wick	–	**/**4 days	–	[229]
CRP, pH, temperature, ionic strength	Thionine‐conjugated AuNPs deposited on laser engraved graphene	Analyte specific, primary and secondary antibodies	Electrochemical	Iontophoresis and microfluidic device	9.6 ng mL^−1^	**/**32 min	9.6–20 ng mL^−1^	[230]
						**/**1 min		
						**/**1 min		
Levodopa	Zeolitic imidazolate/graphene oxide composite deposited on a Au working electrode	Enzymes	Electrochemical	Cotton pad	0.45 µm	7 days, @4 °C**/**40 min	1–95 µm	[231]

^a)^ Stated with no data provided

^b)^ Cortisol and DHEA were detected using two separate biosensors

^c)^ The inclusion of this paper was because the metabolites and electrolytes were being used as stress markers

^d)^ Only IL‐8 was demonstrated to be measured on‐body.

#### 2.9.1 Sweat Sampling

**Figure 4:**
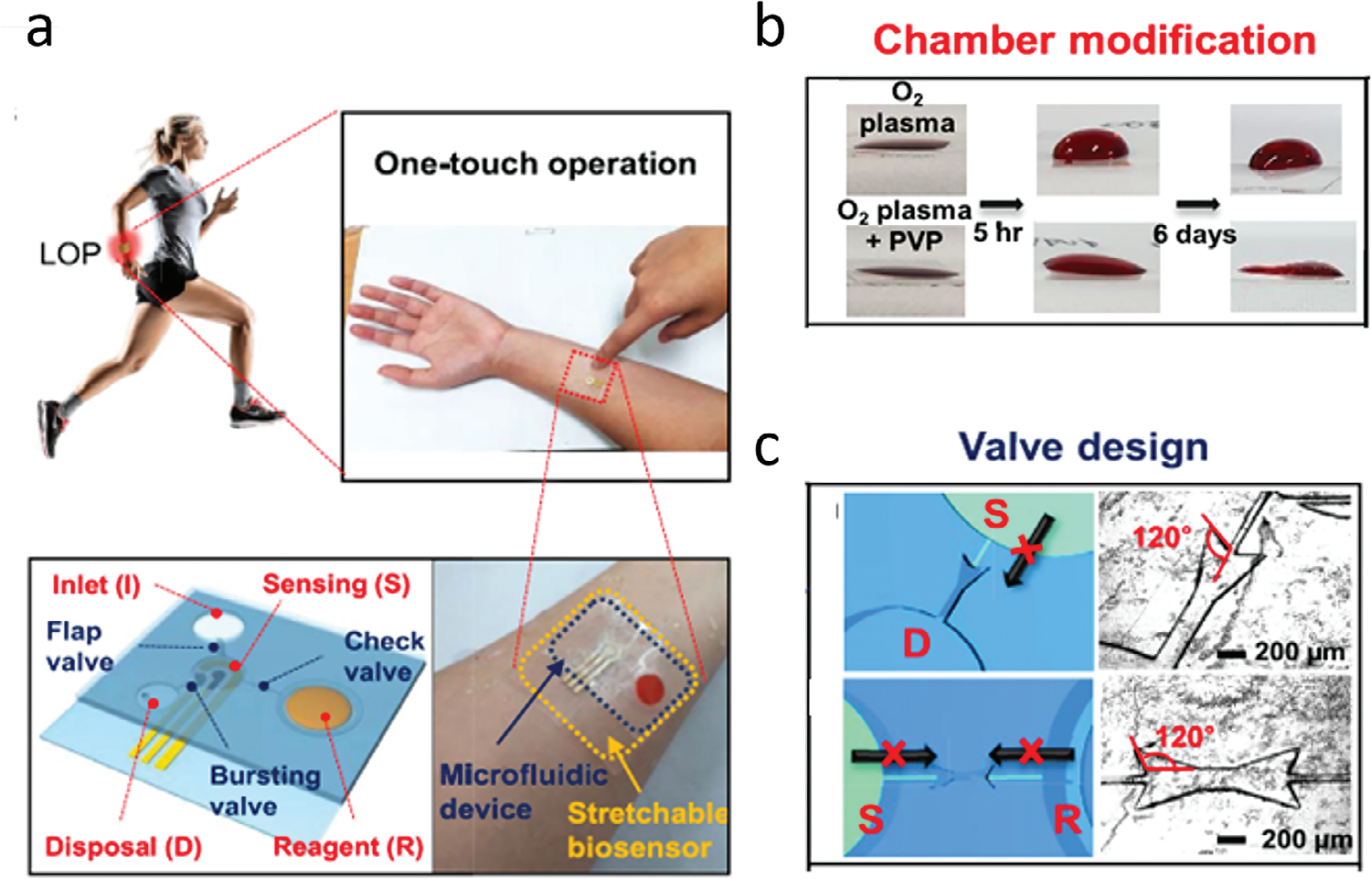
a) The flexible microfluidic patch for detecting sweat cortisol developed by Lee et al. that uses touch actuation for cortisol detection. b) Images showing how plasma treatment after coating the channels with PVP resulted in improved hydrophilic surfaces. c) The bursting valve connecting the sensing chamber (S) to the disposal chamber (D) and the check valve connecting S to the reagent chamber (R) Adapted with permission.^[^ ^205^ ^]^ Copyright 2020, Elsevier.

Typically, WBs for sweat sampling are in the form of a carefully designed soft microfluidic device.^[^ ^232^ ^]^ Flow of sweat into and out of the device is driven partially by the pressure of secretion and partially by capillary forces. Inclusion of an outlet enables the periodic delivery of fresh sample to the active layer. Careful consideration of the patterning of the microfluidic channels and reservoirs, their inner surfaces, the inclusion of valves, and the material used for the device is needed to ensure a consistent quantity of fresh sample fluid is delivered to the active layer. For example, Lee et al. presented a wearable biosensor for the immunodetection of cortisol in sweat that used a carefully designed microfluidics network to deliver sweat to a reaction reservoir (**Figure** **4**a).^[^ ^205^ ^]^ The authors coated microfluidic channels with polyvinylpyrrolidone (PVP) then treated them with oxygen plasma to increase the hydrophilicity of the surfaces (Figure 4b). Doing so facilitated flow of sweat into and through the device. They used valves to control the flow of sweat from chamber to chamber (Figure 4c). They placed a bursting valve to prevent sweat in the sensing chamber from overflowing into the disposal chamber during incubation. They used a check valve between the sensing and reagent chambers to prevent sweat flow between the two chambers unless the user presses the button to deliver the reagent to the sensing chamber.

A caveat to using soft microfluidics alone is the need for ample amounts of fluid to enter the device, such that the volume and flowrate of the fluid passing over the sensor is consistent and continuous. Often when testing these WBs, the subject is instructed to exercise to generate copious amounts of sweat. Having the client exercise every time a sample is needed is impractical for continuous monitoring throughout the day and would be incompatible with most modern lifestyles.^[^ ^233^ ^]^ This rationale has motivated researchers to incorporate hydrophilic polymeric materials for the effective wicking of passively secreted sweat (aka background sweat).^[^ ^234^ ^]^ Prasad's group has consistently demonstrated WSS designs that used a nanoporous polyamide membrane, to continuously wick passively secreted sweat and deliver it directly to the active layer.^[^ ^14^, ^210^, ^211^, ^213^, ^226^, ^233^, ^235^, ^236^, ^237^ ^]^ In other studies,^[^ ^238^, ^239^ ^]^ using room temperature ionic liquids, the group was able to enhance antibody stability in sweat thus allowing for longer monitoring times. Using this wicking method, the group, on separate occasions, managed to continuously monitor the accumulation of cortisol at the active layer up to times of 4,^[^ ^233^ ^]^ 8,^[^ ^211^ ^]^ 9,^[^ ^237^ ^]^ and, recently, 48 h.^[^ ^229^ ^]^

Despite these achievements, the primary issue with wicking is the absence of flow control and the mixing of old and fresh sweat.^[^ ^234^ ^]^ With the exception of electrolytes and certain metabolites,^[^ ^240^ ^]^ this is a minor issue for those smaller biomarkers that are expected to be independent of the sweat rate.^[^ ^190^ ^]^ However, larger, more hydrophilic biomarkers are expected to be more dependent on the sweat rate.^[^ ^130^, ^192^ ^]^ The rate of sweat secretion varies from individual to individual and from sweat gland to sweat gland,^[^ ^241^ ^]^ and is dependent on physiological factors like hydration level, physical health, and mental state as well as environmental factors like temperature and humidity. As such, for NPBs that are expected to change with sweat rate, sensors for sweat rate should be included like what has been done with WSSs that monitor metabolites and micronutrients.^[^ ^242^, ^243^, ^244^ ^]^

The ideal WBS for sweat sampling should be able to account for variable factors in sweat sampling if its purpose is to be used for continuous monitoring in daily life. Toward this goal, sampling methods that integrate electrodes for iontophoresis, osmotic pumps,^[^ ^245^, ^246^, ^247^ ^]^ or absorbent materials with soft microfluidics are more suitable.^[^ ^234^, ^248^ ^]^ Of these, iontophoresis appears to be most promising. Ionotophoresis for sweat induction relies on the delivery of cholinergic agents into the dermis to stimulate sweat production. Because the goal of iontophoresis for sweat induction is drug delivery and not fluid extraction, like how it is used for ISF extraction, lower operating voltages can be used.^[^ ^190^, ^249^ ^]^ Thus, the issue of skin damage is mostly avoided. However, there are still issues that prevent the method from being widely used in practice.^[^ ^250^ ^]^ Nevertheless, the recent publications by Tu et al.^[^ ^230^ ^]^ and Xu et al.^[^ ^13^ ^]^ demonstrated that iontophoresis was a feasible method for continuous sweat sampling.

Aside from issues with sweat rate variability, other challenges regarding wearable sweat sensing are associated with sweat's composition. First, because biomarkers found in sweat arrive from dermal ISF, they are subjected to a filter effect, which further dilutes their concentrations. Second, the pH and salinity of sweat are more variable than in ISF. These variations can impact not only the rate of sweat secretion, but also its composition.^[^ ^234^ ^]^ Additionally, these changes may impact how the analytes interact with their biorecognition elements.^[^ ^12^, ^251^ ^]^ Therefore, depending on the analyte(s) and choice of detection method, WSSs should be designed with contingency sensors that allow for corrections due to changes in pH and salinity. Finally, the proximity of sebaceous glands to eccrine sweat glands poses a possible source of contamination.^[^ ^252^ ^]^ Compared to the face and scalp, the densities of sebaceous glands on the arms and legs are significantly lower.^[^ ^253^ ^]^ However, the role of the endocrine system in the secretion of sebum adds an unexplored source of variability in sweat sensing of NPBs.^[^ ^254^, ^255^ ^]^ Considering recent publication by Sarkar et al. on the classification of lipid biochemical markers in sebum using mass spectrometry for the diagnosis of Parkinson's disease,^[^ ^256^ ^]^ it may be worth investigating the partitioning of lipophilic SBMs, like cortisol, between sebum and sweat, both of which would have considerably different fluidic properties that may impact their transport into the device.

#### 2.9.2 Sensing SBMs with Wearable Sweat Sensors

##### Biorecognition Elements

The selection of a biorecognition element is guided by four primary factors: selectivity, reproducibility, reusability, and sensitivity. The biorecognition element is the component of the biosensor responsible for ensuring that the measured signal is specific to the target analyte. Cortisol is non‐electroactive, therefore detection relies on affinity‐based recognition. Affinity‐based recognition requires the target analyte to have a high binding affinity toward a structural motif of the biorecognition element. So far, antibodies, aptamers, and molecularly imprinted polymer (MIP) membranes have been used for the selective detection of cortisol with wearable sweat sensors.^[^ ^251^ ^]^

Antibodies are the most frequently employed biorecognition elements due to their high selectivity and specificity, which arise from the unique binding between antibodies and antigens. These protein‐based elements are commonly used in immunoassays, where they enable optical or electrochemical detection. Recent advancements have enhanced antibody specificity through modifications with nanomaterials, further reducing cortisol cross‐reactivity.^[^ ^208^ ^]^

**Figure 5:**
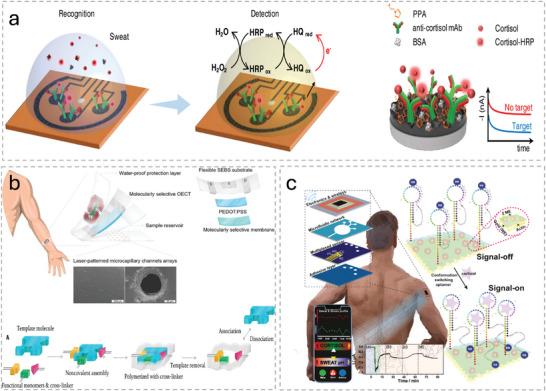
a) The immunosensor developed by Torrente‐Rodriguez et al. that detected cortisol by exploiting the competitive binding between cortisol‐conjugated HRP and sweat cortisol with an anticortisol mAb. Adapted with permission.^[^ ^208^ ^]^ Copyright 2020, Cell Press. b) The wearable cortisol sensor developed by Parlak et al. and the design concept of the MIP biorecognition element. Adapted with permission.^[^ ^10^ ^]^ Copyright 2018, American Association for the Advancement of Science. c) A schematic of the biosensor developed by Singh et al. that used methylene blue‐functionalized aptamers conjugated to gold for cortisol capture and detection. Adapted with permission.^[^ ^224^ ^]^ Copyright 2023, Elsevier.

One notable technique, studied by Torrente‐Rodriguez et al.,^208^ involved competitive binding on an antibody‐modified graphene electrode surface. In this method, sweat cortisol and HRP‐labeled cortisol competed for binding sites, with the reduction of hydrogen peroxide generating a cathodic current. The recorded current was inversely proportional to the cortisol concentration: higher cortisol levels lead to less binding of HRP‐labeled cortisol, resulting in a lower current, and vice versa. The presence of antibodies in this system significantly enhanced specificity and sensitivity toward cortisol, even in the complex mixture of molecules found in sweat (**Figure** **5**a). However, a significant challenge remains: the limited reusability of antibody‐based sensors, which is critical for continuous and frequent monitoring. This aspect is currently the subject of extensive research.^[^ ^208^ ^]^

MIPs are polymer matrices formed in the presence of a target molecule, which, once removed, leaves behind a cavity specific to that target. The specificity of MIPs is determined by noncovalent bonding, electrostatic interactions, and the size and shape of the cavity, all of which can be optimized by selecting the appropriate monomers, crosslinkers, and target molecules, as depicted in Figure 5b, MIPs are more stable at room temperature compared to antibodies and aptamers and offer the advantage of reusability, making them suitable for frequent monitoring. MIPs because of the cavities are less susceptible to reactivities with other ions or bigger biomolecules that could be in abundance in body fluids like sweat.^[^ ^10^ ^]^ Additionally, MIPs are cost‐effective, and their specificity for cortisol is enhanced by the tailored cavities that capture only the target molecule, making them particularly effective in the complex composition of sweat. In general, if the MIP is directly deposited onto a semiconductor polymer channel in electrochemical biosensors known as organic electrochemical transistor (OECT) (Table 2), due to direct diffusion, the selectivity of the sensor diminishes significantly, so in their biosensor, Parlak et al. came up with a device that uses an inert polymeric membrane that encloses the MIP, which helps to increase the selectivity and sensitivity (Figure 5b).^[^ ^10^ ^]^

Aptamers are short DNA or RNA sequences typically developed using Systemic Evolution of Ligands by Exponential Enrichment (SELEX) to bind to specific bioanalytes. Apatamers offer several advantages over antibodies, including greater stability under varying conditions such as pH and temperature, and the potential ability to be reused without losing specificity or function. The effectiveness of aptamers in biosensors is heavily dependent on the binding configuration and the binding energy of the aptamers with their target. Because aptamers can be designed to target a wide range of bioanalytes, they can be considered more versatile than other affinity‐based biorecognition elements.^[^ ^251^ ^]^

WSSs that use aptamers to target cortisol have most often paired the biorecognition element with a capacitive transduction element (Table 2) like nanoporous semiconducting materials, porous conducting polymers, and electrodes decorated with nanomaterials. Because aptamers are negatively charged, it is reasonable to expect a change in the electronic or ionic capacitance of the transduction element in response to changes in aptamer conformation upon binding with its target. This statement, however, is under the assumptions that the active layer is protected from interfering agents that can alter the surface properties of the transduction element and that the resultant change in aptamer confirmation is sensitive enough to generate a signal discernable from the background. As such, if the generated signal can be more specifically linked to changes in aptamer conformation, the sensitivity and selectivity of the biosensor could be improved. Singh et al. approached this problem by using methylene blue‐labeled cortisol aptamer on a gold electrode in the detection of cortisol in human serum (Figure 5c).^[^ ^224^ ^]^

##### Electrochemical Detection of Cortisol with WSSs

Concerning WSSs that detected SBMs more connected to psychological stress, the majority of those demonstrated in the literature targeted cortisol. Regarding the analytical performance of these WSSs in benchtop experiments, the choices of biorecognition and transduction elements have little impact. All are capable of sensing cortisol within its physiological range (8–140 ng mL^−1^)^[^ ^257^ ^]^ and can distinguish the molecule from structural analogues in artificial sweat or secreted sweat. Cortisol is not electroactive, is lipophilic, and does not have a complimentary enzyme. Therefore, common electrochemical detection methods for small molecules like voltammetry, potentiometry, and enzyme mediated amperometry are not practical, without additional reagents to enhance sensitivity.^[^ ^161^ ^]^ Such was the case for Torrente‐Rodríguez et al.^[^ ^208^ ^]^ To improve the accuracy of their biosensor they included cortisol conjugated to horseradish peroxidase (cortisol–HRP) as a competitive inhibitor of cortisol. When bound to anticortisol antibodies conjugated to the working electrode, HRP reduced hydrogen peroxide in the sample fluid to water when in the presence of the redox mediator hydroquinone. The amount of cortisol bound to anticortisol antibodies would then be inversely proportional to the change in cathodic current due to the oxidation of hydroquinone by HRP.^[^ ^16^ ^]^ Contrary to this approach, the majority of detection strategies rely on the change in impedance at the active layer following the binding of cortisol to either MIP membranes, aptamers, or antibodies. Signal transduction is then performed using an inert conductive material, like gold or graphene, or a semiconductive material, like ZnO. In the former case, the measured current change is due to the change in faradaic impedance (i.e., resistance and capacitance) at the active layer following the binding event. For such strategies, it is common to include an electroactive reporter molecule to enhance the sensitivity. In the latter scenario, changes in current are due to changes in nonfaradaic impedance (i.e., charge capacitance) at or in the semiconductor layer. Such strategies either use a porous semiconductor material, an OECT,^[^ ^258^ ^]^ or an electrolyte‐gated field effect transistor as the transduction element.^[^ ^259^ ^]^ Most of these devices have been thoroughly reviewed in the recent publications by Karuppaiah et al.^[^ ^16^ ^]^ and Ok et al.^[^ ^260^ ^]^

Validating the stability of an electrochemical biosensor is essential for continuous monitoring applications. Cortisol, cytokines, and other SBMs follow either circadian or ultradian rhythms. Their concentrations are continuously changing throughout the day. These natural variations along with potential fluctuations due to exogenous factors may contribute to measured signals, hence why long‐term sensor stability is of utmost concern for continuous monitoring applications. A typical method for assessing long‐term stability is to determine the storage lifetime, which basically involves periodically checking the sensor's signal output in response to a standard amount of analyte over an extended period. However, the storage lifetime does not adequately account for real‐life factors that would impact sensor performance in continuous monitoring applications. A possible better measurement of long‐term stability would be to simulate sweating by using a microfluidics system to that continuously flows artificial sweat spiked with interferents over the biosensor. Tu et al.^[^ ^230^ ^]^ performed a similar test in their recent paper detailing a wearable device for monitoring CRP. The group used a syringe pump to evaluate how the flow rate and ionic strength of CRP‐spiked PBS would impact the measured signal. They tested flow rates that would equate to high sweat rates,^[^ ^192^ ^]^ thereby essentially testing the sensor's performance in a worst‐case‐scenario. Many of the studies tabulated in Table 2 provided a satisfactory alternative to such a quality test by demonstrating continuous operation of the WBSs while on‐body. However, few provided data showing their WBS was still comparable to standard assays during and at the end of continuous operation.^[^ ^213^, ^229^ ^]^

##### Optical Detection of Cortisol with WBSs

**Figure 6:**
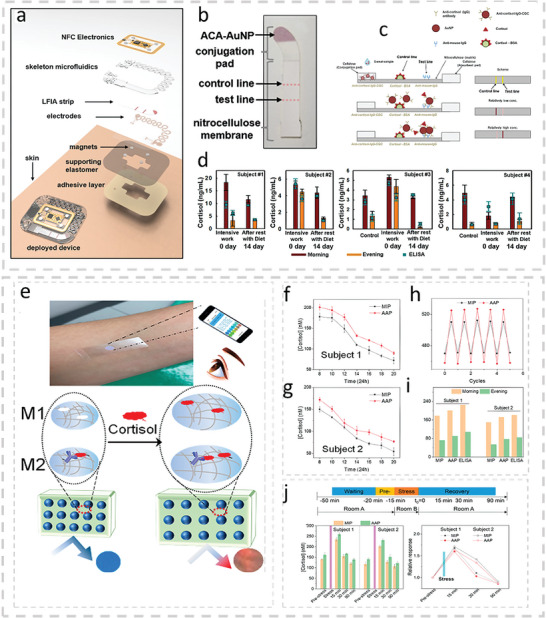
a–d) The multiplex WSS for detecting cortisol, glucose, and ascorbic acid developed by Kim et al. The cortisol LFA strip is incorporated into the device (a) microfluidics. (c) A schematic depicting the detection mechanism of the cortisol LFA. (d) Data showing the WSS was capable of capturing the circadian variation of cortisol with accuracy comparable to a standard ELISA. Adapted with permission.^[^ ^223^ ^]^ Copyright 2020, PNAS. e–j) The WSS developed by Qin et al. that uses photonic hydrogels for cortisol detection. (e) A picture of the final sensor worn on a subject's arm along with an illustration depicting the detection mechanisms of the device that used the MIP method (M1) and the antibody–antigen polymer (AAP) method (M2). (f–g) Subject data showing both devices capable of monitoring sweat cortisol over a 4 h period. h) Measured absorbance of the photonic crystals following a cycle of before detection, after detection, and after washing. j) Monitoring results during the Trier Social Stress Test showing both devices capable of detecting variations in sweat cortisol throughout the test. Adapted with permission.^[^ ^19^ ^]^ Copyright 2023, American Chemical Society.

Few WSSs that target cortisol use optical detection methods. Some main advantages of using optical detection methods instead of electrochemical methods are not needing sophisticated electronics and, consequently, not needing a power source. A downside is that many require additional equipment for detection and analysis. However, smartphone technology has enabled quick and easy analysis of colorimetric assays. Spurred by the involvement of cortisol, ascorbic acid (vitamin C), and glucose in the body's stress response, Kim et al.^[^ ^223^ ^]^ developed a multiplex WSS that relied entirely on optical‐based detection methods (**Figure** **6**a–d). Cortisol was detected with an integrated, lateral flow immunoassay (LFI) situated near the end of a serpentine microfluidic channel. Cortisol in sampled sweat would bind to anticortisol antibodies conjugated to AuNPs as the sweat flowed through the LFI. At the test strip, the anticortisol antibody would bind with a secondary antibody specific to the primary's isotype (IgG). Increasing concentrations of analyte led to a perceptible color change that could be detected using a smartphone app. Fluorometric detection of glucose and ascorbic acid proceeded by enzymatic oxidation and ended with reduction of a fluorescent probe. The resulting change in fluorescence was detected using a special apparatus, with integrated excitation and emission filters, that could be attached to a smart phone.

A drawback to the design of Kim et al. was the LFI, which would need continuous replacing. Qin et al.^[^ ^19^ ^]^ developed two wearable photonic hydrogels for the continuous monitoring of cortisol in sweat (Figure 6e–j). A notable feature of both devices was their reusability after washing to remove bound cortisol. The first design relied on an MIP for selective detection of cortisol. The second relied on antibodies noncovalently bound to the hydrogel matrix. The first design was fabricated by first mixing polystyrene colloidal photonic crystals (CPCs) with polyvinyl alcohol (PVA). Afterward, the polymers were chemically crosslinked in the presence of cortisol to form the MIP template and photonic hydrogel. The second design was fabricated by mixing the CPCs with PVA, then adding anticortisol bound to BSA‐conjugated cortisol before chemically crosslinking. Both designs relied on local volume changes in the photonic crystal to generate changes in refractive index and color of the bulk photonic hydrogel. In the case of the first design, volume changes were induced through cortisol binding with the MIP template. For the second design, volume changes were induced through dissociation of anticortisol from BSA‐conjugated cortisol in favor of binding to free cortisol. The resulting color change would then be measured using a smartphone app. The group demonstrated that both devices were capable of continuously monitoring cortisol in secreted sweat in response to psychological stress induced by the Trier Social Stress Test (TSST), which is explained in greater detail later in this review.

Few WSSs have targeted larger (i.e., high molecular weight) SBMs. These SBMs are chemically more complex than cortisol and other smaller SBMs, which translates to a greater tendency to form nonspecific interactions. Moreover, they exist at low concentrations in serum that translate to even lower concentrations in the other peripheral biofluids. These qualities of larger SBMs make detecting them a challenge especially in sweat. So far, most large SBMs targeted by WSSs have been cytokines, none of which for stress‐monitoring applications.

**Figure 7:**
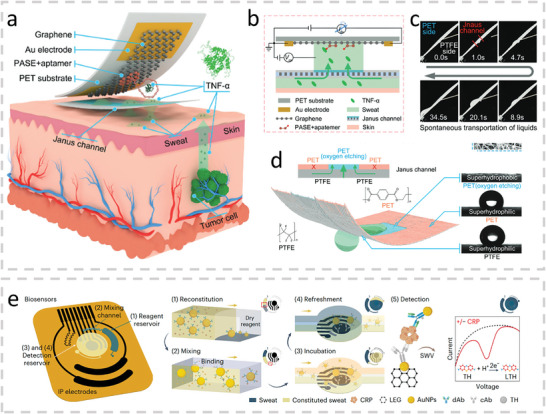
a–d) The WSS developed by Huang et al. for the detection of TNF‐α. (a) An illustration of the wearable patch showing the principal components. (b) A schematic showing transport of sweat to the active layer of the graphene field effect transistor by the Janus channel. (c) Images demonstrating the rate of transport of water through the channel. Adapted with permission.^[^ ^228^ ^]^ Copyright 2023, Wiley‐VCH GmbH. (d) An illustration of the Janus channel showing the principal polymer components. e) An illustration of the WSS developed by Tu et al. for detecting CRP along with a schematic showing the reconstitution of AuNP probes, their delivery to the sensor, and detection of CRP. Adapted with permission.^[^ ^230^ ^]^ Copyright 2023, Springer Nature.

Two recent papers that targeted cytokines took unique approaches to dealing with the challenges familiar to detecting biomacromolecules. Huang et al.^[^ ^228^ ^]^ combined a Janus channel with a graphene field effect transistor into a wearable patch for detecting TNF‐α in sweat (**Figure** **7**a–d). The Janus channel consisted of a polytetrafluoroethylene/polyethylene glycol terephthalate (PTFE/PET) substrate with the PTFE side interfaced with the skin. Oxygen plasma etching of a defined area of the PET converted that area of the material from superhydrophobic to superhydrophilic. When sweat contacts the superhydrophobic side, there was a Laplace force that transported the sweat across the hydrophobic layer into the hydrophilic layer in a unidirectional manner. Interfaced with the hydrophilic layer was a graphene layer functionalized with a TNF‐α‐specific aptamer, which acted as the channel of the electrolyte‐gated field effect transistor. Capacitive changes at the channel due to TNF‐α binding with its aptamer resulted in sensitive changes in measured current, due to changes in channel transconductance, proportional to the amount of TNF‐α bound. The authors evidenced that the micropores of the Janus channel serve to filter large debris from the sweat sample, thereby concentrating the amount of TNF‐α in the sweat sample.

Tu et al.^[^ ^230^ ^]^ approached the problem by combining a traditional sandwich assay with a microfluidic device and iontophoretic module (Figure 7e–h). Sweat stimulated by the iotophoretic delivery of carbachol flowed into the reagent reservoir of a microfluidic device. The sweat reconstituted AuNPs conjugated with the redox molecule thionine and an anti‐CRPα detection antibody. CRP in the sweat sample bound to the AuNPs before the mixture reached the detection reservoir. Here, TNF‐α bound to a secondary capture antibody conjugated to AuNPs deposited on laser engraved graphene. The current generated from the oxidation of thionine was proportional to the amount of TNF‐α captured. Using this method, the group improved both the sensitivity and selectivity of the biosensor.

#### 2.9.3 Multiplex Detection of SBMs with WSSs

The applicability of these WBSs in neuropsychiatric medicine is limited, unless more than one SBM is targeted. There are several publications demonstrating multiplex WSSs for on‐body monitoring of SBMs (Table 2). So far, those that target cytokines have done so without additionally targeting cortisol or without the intention of stress‐monitoring applications, instead using them for detecting inflammation for disease states. Those which are designated for stress monitoring applications usually target cortisol in addition to autonomic markers or just autonomic markers. In these cases, the feasibility of the devices for monitoring stress was validated using physical and/or psychological stress tests.

**Figure 8:**
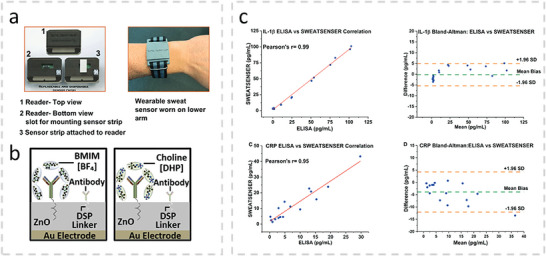
a) The WSS used by Jagannath et al. b) An illustration of the biosensor's active layer. Adapted with permission.^[^ ^238^ ^]^ Copyright 2018, Elsevier. c) Data showing comparisons of the IL‐1β and CRP detection capabilities of the multiplex SWEATSENSER and standard ELISA's. Bland Altman analyses (right) indicated no significant statistical difference between the standards and the device. Adapted with permission.^[^ ^227^ ^]^ Copyright 2021, Oxford Academic.

Jagannath et al.^[^ ^227^ ^]^ repurposed the WSS technology (**Figure** **8**) their lab previously developed for the multiplex detection of CRP and IL‐1β in eccrine sweat for monitoring flare ups in those suffering from irritable bowel disease.^[^ ^211^, ^238^, ^261^ ^]^ The device relied on the passive sampling of sweat using a hydrophilic polymer membrane. CRP and IL‐1β were detected using monoclonal antibodies stabilized with an RTIL and conjugated to a nanoporous ZnO semiconductor. The WSS, dubbed “SWEATSENSER” was used to establish excellent correlations (Pearson's *r* = 0.99 for IL‐1β and *r* = 0.95 for CRP) between sweat and serum concentrations of the two cytokines. The group reported that the biosensor was capable of continuously monitoring IL‐1β up to 30 h. Data showing continuous monitoring data for CRP were not provided. However, in the paper of Jagannath et al.,^[^ ^262^ ^]^ where the device was used for the multiplex detection of CRP, interferon inducible protein (IP‐10), and tumor necrosis factor‐related apoptosis‐inducing ligand, they demonstrated that detection of CRP in sweat with SWEATSENSER was reproducible. This is currently, to the best of our knowledge, the only study that reported the multiplex detection of two validated protein SBMs using a WSS.

**Figure 9:**
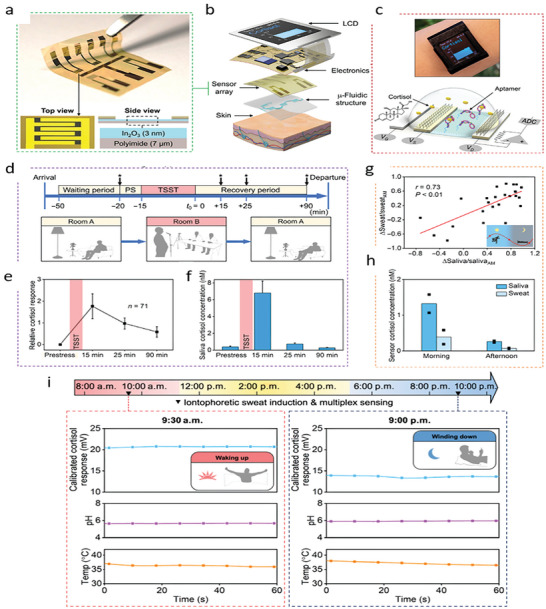
Smartwatch developed and used by Wang et al. for the multiplex sensing of cortisol in sweat, temperature, and sweat pH. a) A photograph of the FET array and top‐view and side‐view schematics. b) A schematic showing the essential components of the smartwatch. c) (Top) A photograph of the smartwatch worn on someone's wrist. (Bottom) A schematic illustration showing the key components of the cortisol‐sensing, electrolyte‐gated FET. d) An illustrated summary of the TSST. e) A plot showing the relative change in average salivary cortisol levels of 71 subjects during the stages of the TSST using a benchtop version of the biosensor. f) A bar chart showing the average salivary cortisol levels of a representative subject during the stages of the test. g) ELISA measurements were used to construct this correlation between cortisol concentrations in saliva and sweat collected at night and morning (*n* = 17) to establish a relation between sweat and saliva cortisol. h) Morning and afternoon measurements of a representative subject's saliva and sweat cortisol levels using the cortisol detecting FET. i) Real‐time monitoring of sweat cortisol, sweat pH, and skin temperature of a healthy subject at two different time points with the smartwatch. Adapted with permission.^[^ ^11^ ^]^ Copyright 2022, American Association for the Advancement of Science.

The wearable smartwatch (**Figure** **9**a–c) designed, developed, and validated by Wang et al.^[^ ^11^ ^]^ incorporated a multiplex sensor that used an electrolyte‐gated field‐effect transistor (FET) array coupled with a custom‐built source measurement unit for the simultaneous, real‐time monitoring of sweat cortisol, sweat pH, and skin temperature. The cortisol sensitive FET used in‐house‐designed, cortisol‐targeting aptamers. A notable experiment described in the paper, used to validate the analytical capabilities of the sensor, involved the Trier Social Stress Test.^[^ ^263^ ^]^ The group measured cortisol levels in the saliva of participants (*n* = 71) of the TSST before test administration, 15 min after administration, 25 min after administration, and 90 min after administration (Figure 9d) using a benchtop version of their biosensor. They found that there was a large increase in salivary cortisol 15 min after the TSST (Figure 9e,f) was comparable to that measured using ELISA and liquid chromatography in tandem with mass spectroscopy. As a demonstration of the watch's on‐body capabilities of multiplex detection of cortisol, pH, and temperature in sweat, sweat from a human subject was iontophoretically stimulated with a separate commercial device then collected by the microfluidics module. Measurements with the device were correlated with salivary cortisol levels measured using an ELISA assay (Figure 9g,h). Real‐time analysis occurred over the course of 1 min at 9:30 am and 9:00 pm. The cortisol response, pH, and temperature data indicated that the measured quantities were constant during the 1 min sampling time and that nighttime cortisol levels of the subject were lower than their morning cortisol levels (Figure 9i). This result is consistent with the circadian cycle.

**Figure 10:**
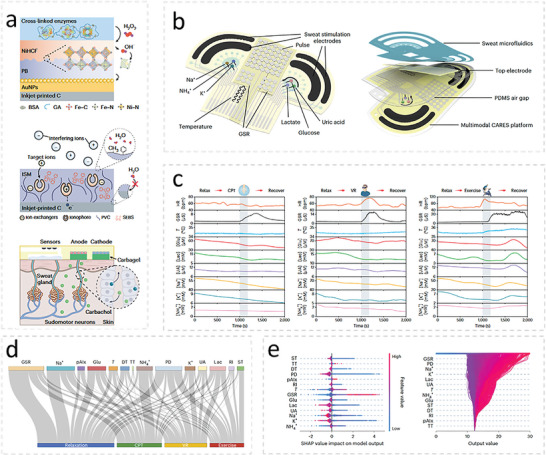
a) Schematics depicting the detection mechanisms for the metabolites (top) and electrolytes (middle) in addition to an illustration (bottom) illustrating the iontophoretic delivery of carbachol for sweat gland stimulation. b) A schematic of the final device. c) Subject data showing the WSS capable of monitoring the six analytes during administration of the CPT, VR test, and exercise. d) A diagram showing the Shapley additive explanation (SHAP) analysis of the data collected by the individual sensors. The diagram illustrates the relative contribution of each analyte to classifying a stressor. e) Plots depicting the SHAP analysis of data collected during administration of the STAI‐Y questionnaire. The plot on the left shows the relative contribution of the analytes to determining the state anxiety level. The plot on the right depicts the impact of the variables on the machine learning model's final output of state anxiety level. Adapted with permission.^[^ ^13^ ^]^ Copyright 2024, Nature Electronics.

Different stressors are processed differently in the CNS. Being able to differentiate between multiple stressors would be valuable for delineating the contributions of physical stressors to the stress response from those of psychological stressors, and consequently aid with biomarker discovery. Xu et al.,^[^ ^13^ ^]^ in their recent publication, developed a reproducible, multimodal WBS that used machine learning to differentiate different sources of stress (**Figure** **10**). The WBS consisted of an iontophoretic module that used carbachol to stimulate sweat secretion; enzymatic biosensors for detecting glucose, lactate, and uric acid; ion‐selective electrodes for measuring Na^+^, NH_4_^+^, and K^+^ ions; a capacitive pressure sensor for measuring arterial pulse; temperature sensors for measuring skin temperature; and Ag electrodes for measuring skin conductivity. The device was demonstrated capable of monitoring all analytes over a 24 h period with minimal signal drift. Participants were exposed to three different stress tests: the cold pressor stress test (CPT), a virtual reality test where participants were asked to compete against one another, and exercise. Before and after each test, participants were asked to fill out the State‐Trait Anxiety Inventory Form‐Y (STAI‐Y) questionnaire to gauge the participants’ anxiety levels. The data collected from the three experiments were fed to a machine learning algorithm that was instructed to differentiate stress from relaxation, classify each stressor, and evaluate anxiety levels. The model was able to distinguish stress from relaxation with 99.2% accuracy, to classify the three stressors with 98% accuracy, and to predict state anxiety levels with 98.7% confidence. Interestingly, they found glucose, skin conductivity, Na^+^ concentration, and pulse were the most important variables for differentiating the stressors. Skin conductivity, pulse, Na^+^, K^+^, and lactate were most important for predicting anxiety levels.

## 3 Outlook and Perspective

The significance of conventional biomarkers for aiding treatment has been studied by health professionals and engineers. Point of Care Testing is an important tool in the field of behavioral health, considering its utility as a patient‐centered healthcare device. However, the demand for an objective assessment in clinical psychiatric diagnosis and treatment does pose certain challenges since a biomarker can be clinically useful only if it is specific to a disorder, reproducible, and accurate in both predictability and assessment. Consequently, the sensing technology is also required to be high end beside it being reliable, valid, and economically feasible in nature. Even though, several biomarkers have also been validated clinically to be associated with various neuropsychiatric conditions, they still have a long way to go to be implemented in clinical practice. It is the lack of clinical validation of certain novel biomarkers in large populations and the unavailability of a commercial POC testing device in the clinic for detecting the biomarkers that raises the question about both the clinical significance as well as its usability in health care settings to clinical practitioners—a rigorous interdisciplinary research being the only bridge to this barrier. Thus, in addition to the challenge of clinically validating these new biomarkers, a device specific for detecting each biomarker, needs to be designed and fabricated.

Wearable biosensors represent an impending paradigm shift in neuropsychiatric healthcare if designed and implemented with function in mind. The time and situational dependence of the development and persistence of many stress‐related NPIs stands to benefit from a device capable of continuously monitoring, in real‐time, changes in biochemical markers that can elucidate possible etiologies, monitor treatment efficacy, or even just determine possible stressors. In this penultimant section, we discuss future directions for wearable biochemical sensing for addressing some of the fundamental challenges of detecting SBMs for neuropsychiatric applications regarding continuous monitoring, SBM heterogeneity, and multiplex detection.

### 3.1 Future Strategies for Wearable Biosensors Targeting SBMs

Despite cortisol's lack of specificity, irregularities in its secretion can be reliably connected to psychological stress. Cortisol, therefore, is still expected to be an essential target of future biosensors intended for neuropsychiatric applications. Sweat is expected to remain the preferred biofluid for cortisol detection because sweat can be sampled noninvasively and continuously. Additionally, electrochemical detection of the hormone will likely be the preferred detection strategy due to the analytical success of current WBSs that target it. However, there remain challenges that impede adaptation of these WBSs into more continuous‐monitoring formats. The capability to measure cortisol levels continuously is vital for enhancing our understanding of the body's stress response, providing insights into stress‐related disorders such as anxiety and depression. Future strategies for cortisol detection with WBSs should focus on improving the contents of the active layer to improve monitoring times and/or sensor reusability. 

Antibody‐based sensors are among the most widely used due to their high specificity and sensitivity. However, these sensors have significant drawbacks, including cross‐reactivity, stability issues, and high costs, that may impact the performance and implementation of future WBSs for cortisol detection. To enhance stability, various stabilizing agents, such as trehalose and sucrose, are being explored for antibody storage. Ionic liquids such as cholinium‐based ionic liquids have also seen success in recent years for improving antibody stability during storage and sensing.^[^ ^239^, ^264^ ^]^ Additionally, recombinant antibody fragments like scFv and Fab have demonstrated greater stability and cost‐effectiveness. Beyond antibodies, MIPs and aptamers offer alternative approaches. While these alternatives present challenges in selectivity and binding affinity, they have the advantages of possible reusability and anti‐biofouling potential. There are reports of reusable aptasensors that use buffers to regenerate the unbound state with only a slight loss in binding efficiency.^[^ ^265^, ^266^ ^]^ Aptamers can also be functionalized with zwitterionic peptides to prevent the absorption of biomolecules on the surface.^[^ ^267^, ^268^ ^]^ MIPs, in particular, can achieve better selectivity through optimized polymerization conditions. More studies need to be done on this in the future. Improving the stability of electrochemical cortisol sensors is another critical area. The use of high‐quality, stable electrode materials such as gold and graphene can enhance both stability and sensitivity.^[^ ^16^ ^]^ Addressing factors like biofouling, where samples accumulate on the sensor surface, is also important; functionalizing the sensor surface with hydrogels or other types of polymers can help mitigate this issue. Long‐term stability studies on electrochemical sensors, including aging tests, are essential. Scientists are trying to come up with alternatives for anti‐biofouling to increase longevity, one technique reported was using an oil membrane based aptamer that can help to separate hydrophobic analytes from the entire solution.^[^ ^269^ ^]^ Others used block copolymers, similar in concept to the zwitterionic peptides conjugated to the aptamers, to prevent biomolecules from absorbing onto the electrode surface.^[^ ^270^ ^]^

Detecting cortisol is challenging due to its low concentrations in body fluids and the presence of similar compounds like cortisone and corticosterone. Continuous monitoring in sweat is particularly complicated by the low volume of sweat released and environmental factors such as humidity and temperature. High humidity can dilute sweat samples, while elevated temperatures can concentrate them, potentially affecting baseline measurements and complicating continuous monitoring. Effective sweat sampling is therefore paramount to ensure measurements are consistent on a day‐to‐day basis. Currently, iontophoretic delivery of sweat stimulants is the most viable option for controlling the amount of sweat that enters the WBS. The technical complexity of the final device however begs the question of the feasibility of ISF sampling WBSs. Of course, there will also be some variability of ISF. However, a microneedle‐based platform would not necessarily require a strategy for maintaining flow across the sensor and therefore would be comparatively simpler in design.

In addition to cortisol, it is crucial to develop strategies for sensing more complex SBMs. The larger size and multiple binding sites of these biomarkers pose challenges for the specificity of biosensors. However, the ability to continuously monitor changes in multiple SBM concentrations remains a critical requirement. Complex SBMs like BDNF are traditionally detected using techniques such as sandwich assays, which employ multiple anti‐mBDNF antibodies with very low cross‐reactivity. However, the need for two antibodies is impractical for continuous, long‐term monitoring applications. Aptamers and MIPs therefore have an advantage in this respect because both can be designed specific to biomacromolecules like BDNF. Several biosensors have been developed for BDNF detection, including: 3D aptasensors based on biolayer interferometry technology for early detection of glaucoma and BDNF and MIP‐based synthetic receptors. In the first case, Gao et al. used a dimeric aptamer to detect BDNF in serum using interferometry.^[^ ^271^ ^]^ For the latter biosensor, the researchers were able to distinguish the BDNF from other molecules like CDNF, MANF, and mCD48 (which is a similar sized macromolecule). Unfortunately, with each use, the response reduced greatly which is not good for the reusability of the sensor. More research needs to be done on the anti‐biofouling and continuous use of this type of biosensor for such big molecules.^[^ ^272^ ^]^ Consequently, the issue of biofouling of the sensor becomes more prominent. The inclusion of size‐excluding membranes is no longer viable. Given the complexity of these molecules, effective strategies must be developed to prevent biofouling on the sensor surfaces, else alternative targets should be explored.

### 3.2 MicroRNA as Emerging Biomarkers of NPIs

Effective use of cortisol detection, and, subsequently, effective use of stress‐monitoring with WBSs for neuropsychiatric applications require simultaneous targeting of cortisol alongside other SBMs. Even so, compared to cortisol, these other SBMs like BDNF, IL‐6, TNF‐α, etc., are chemically more complex, are generally available at lower concentrations, and also lack specificity to any NPI. Alternative analytes, found in the peripheral, that can be more easily detected or can be more easily correlated with dysfunctions in the CNS would be beneficial. Targeting microRNAs is a possible future alternative to targeting typical SBMs. MicroRNA (miRNA) are noncoding oligonucleotides of 19–25 bases in length. Generally, miRNAs can be found throughout the body and participate in post‐transcriptional processes by interacting with messenger RNA to influence gene expression. The influence of epigenetics on the pathophysiologies of NPIs is an active field of research. Thus far, large sets of miRNAs have been implicated in the pathophysiology of NPIs such as schizophrenia, MDD, anxiety disorders, Parkinson's, Alzheimer's, and PTSD from postmortem, rodent, and clinical studies.^[^ ^273^, ^274^, ^275^, ^276^, ^277^, ^278^ ^]^ miRNAs found circulating in peripheral biofluids that participate in the regulation of neuroinflammation, neuroplasticity, and neurotransmission are of particular interest to biosensing as they can be directly linked back to dysfunctions of the CNS. Interestingly, some miRNAs seem to be specific to certain NPIs, though, like with the other SBMs, there is a lack of large‐scale clinical validation to confirm them as satisfactory targets. Unfortunately, unlike the other SBMs, generally, whether these miRNAs can be found in biofluids like saliva, tears, sweat, or ISF is an ongoing area of research. Whether miRNAs could be detected in sweat has only recently been reported on. Nonetheless, miRNA markers are ideal targets for biosensors that employ aptamers as biorecognition elements. Unlike with cortisol, or any other non‐nucleotide containing analyte, the base–base pairing between target miRNAs and their complimentary aptamer will assure a specific biosensor.

### 3.3 Using Machine Learning alongside Multiplexation

The goal of using big data for improving clinical and patient outcomes in neuropsychiatric healthcare is to introduce more quantitative measures to diagnostic and prognostics practices while also offsetting the significance of interindividual variability between patients.^[^ ^279^ ^]^ Integration of machine learning/A.I. into WBSs will be crucial for data processing, data interpretation, data classification, and identifying hidden correlations regardless of whether the biosensor is multiplex, however, it becomes ever more crucial with increasing number of analytes. There are already multiple wearable devices demonstrated as effective for monitoring of autonomic markers and behavioral patterns for improving predictive outcomes in neurodegenerative disorders, stress management, and symptom classification for patients diagnosed with NPIs.^[^ ^9^ ^]^ Current devices intended for stress monitoring will often adopt multiplex/multimodal approaches to symptom monitoring and use machine learning for analyzing and interpreting the data.^[^ ^280^ ^]^ The three most common metrics are the autonomic stress markers, HRV, skin conductivity, and skin temperature, each of which can be broken down into submetrics that can be used to train the integrated algorithm. These submetrics are also intended to increase, for example, HRV, and can be broken down into interval between heart beats (NN), the time‐average NN, mean heart rate, and the difference between the longest and shortest NN, to name some. Each submetric are time‐dependent and will contribute to the interpretation and classification of the measured data.^[^ ^281^ ^]^ The raw collected data, based on these metrics, can then be used toward generating a individual or generalized phenotype representative of the disorder.^[^ ^279^ ^]^

Machine learning implementation for continuous monitoring of SBMs for neuropsychiatric applications still faces the challenge of relying heavily on symptomatology for data interpretation. This is not as much of an issue for prognostic applications, especially for neurological disorders where autonomic symptoms can be reliably correlated to specific dysfunctions of the CNS.^[^ ^282^ ^]^ Combining machine learning with WBSs for diagnostics, on the other hand, is still impeded by the heterogeneity of the illnesses and the lack of specificity of SBMs. This last point stipulates the need for more robust biochemical markers for NPIs, but also, does not account for the time‐dependence of SBMs. The circadian and ultradian variations of most SBMs are well known, however, there is a lack of devices capable of capturing these variations and correlating them to specific NPIs. Integration of machine learning with wearable devices capable of continuously monitoring biochemical markers could potentially lead to discovering hidden, time‐dependent correlations that can make standard SBMs more robust. Upton et al., in their recent publication, demonstrated the rhythmic variations of 8 adrenal steroids in 218 volunteers.^[^ ^283^ ^]^ The group used a portable sampling and collection unit that used microdialysis to continuously collect fractions of subcutaneous ISF from patients over a 24 h period. They used liquid‐chromatogrpahy tandem mass spectroscopy to identify and quantify the analytes. An important result of the study was using dynamic markers to establish patterns of healthy variability, which would then be used to identify individuals’ hormone dysregulation represented by disruptions in their circadian rhythm. As such, the purpose of these dynamic markers is analogous to that of the submetrics of HRV. The group did not use ML for data analysis. However, they used a methodology for establishing a defined state of healthy variability similar to wearable stress sensors that target autonomic markers and use integrated ML. As such, it is easy to imagine the potential impact of using ML to analyze such multidimensional data sets.

### 3.4 Clinical Implications

Monitoring daily changes in emotions and behavior, momentary shifts in mood and physical activity, and capturing changes at specific time points in association with environmental factors and life events has long been central to research in social and behavioral sciences and this approach has often been referred to as “burst design.”^[^ ^284^ ^]^ Current clinical methods will either monitor respiratory rate or HRV for the continuous monitoring of stress.^[^ ^285^, ^286^, ^287^, ^288^, ^289^, ^290^ ^]^ Though these wearable devices provide useful information regarding a patient's immediate response to a stressor, they provide little biochemical information or elucidate potential long term impacts. However, continuous measurement of SBMs to track real‐time physiological responses to stimuli, such as stress or drug exposure, requires specialized sensors capable of ongoing monitoring of the biofluids. Point‐of‐care devices that provide point‐like measurements of SBMs are particularly feasible in research involving neuropsychiatric conditions, especially considering the lack of such devices in current standard clinical practice. These devices can be used in clinical settings to measure biomarkers, ensuring that the process aligns with ethical standards while offering practical benefits for patient care. However, they still lack the potential for greater temporal resolution and the convenience of WBSs.

Papers cited in this review as well as others that target autonomic symptoms have proved capable of capturing physiological responses to acute stressors in the form of changes in cortisol, glucose, heart rate, etc. Regarding psychological stress and cortisol detection, the only report cited in this review to test the proposed WBS with the standard Trier Social Stress Test was that of Qin et al. (Figure 6). The chosen sampling period of 15 min was enough to capture the spike in cortisol following test administration; however whether this temporal resolution is enough to be clinically significant remains to be seen. This unfortunately also implies that a POC device that takes point‐like measurements would provide similar temporal resolution of current WBSs for cortisol detection.

Studies on the short‐term effects of stress have shown that stress can lead to significant changes in immune‐protective, immune‐pathological, and immune‐regulatory responses.^[^ ^291^ ^]^ The interaction between stress and immune responses depends not only on the concentration of hormones like cortisol but also on the precise timing of these hormonal changes relative to immune system activation. Hormonal fluctuations due to stress occur over dynamic time courses, with immediate effects that can peak quickly (e.g., cortisol) relative to the delayed cytokine responses that may evolve over hours or days. These factors are critical when assessing how stress affects immune function and highlight the importance of using time‐resolved measurements to capture both the timing and magnitude of hormonal and cytokine changes. However, current biosensors and in vivo measurement techniques often face challenges in providing continuous, real‐time data and distinguishing between physiological and pharmacological levels of biofluids, which complicates the interpretation of immune response dynamics.

Considering the challenge of aligning the temporal resolution with biomarker measurement with biosensors, the clinical condition which has received most highlights has been post‐traumatic stress disorder, where physiological responses can vary greatly over time and continuous monitoring might help as a “biofeedback mechanism” to the person in distress.^[^ ^292^ ^]^ In contrast, conditions like postpartum depression which may have gradual physiological shifts could be benefitted by temporal measurement of SBMss in various ways.^[^ ^293^ ^]^ Suseelan and Pinna,^[^ ^294^ ^]^ in their review paper, also acknowledged the heterogeneity of biomarkers in depression and emphasized the importance of monitoring biomarkers. If biomarkers for clinical conditions like depression or anxiety can be tracked over both continuous time periods (bursts of time) or even cross‐sectionally at specific time points using the adequate device, then it would help prevent misleading conclusions and diagnosis. Ultimately, the ability to match the time resolution of measurements with the underlying physiological processes is critical for both accurate research and effective clinical management of these conditions. Continuous monitoring provides a more accurate representation of dynamic physiological changes, offering potential improvements in diagnosing and treating disorders with complex, time‐sensitive biomarkers.

## 4 Conclusion

The targeting of SBMs in peripheral biofluids with WBSs is positioned to become a robust, ancillary tool in the studying and treatment of NPIs. Though, the wearable biosensing technology thus far has proved capable of reaching the analytical specifications required for sensing trace biochemical markers in peripheral biofluids, the trends in the current technology have diverged from current literature on psychological stress and its connections to NPIs. Too much focus has been given to the noninvasive detection of changes in cortisol, which is too nonspecific a marker of psychological stress to be detected alone, or markers of autonomic dysfunction. For WBS to be relevant in clinical practice, they clearly must at least be multiplex. Even though multiplex devices are becoming more prevalent, they are not targeting biomarker combinations demonstrated or evidenced in the literature to be more robustly linked to NPIs. Moreover, the majority of devices have thus far have focused on sweat sampling rather than exploring other viable peripheral fluids. Consequently, the lack of diversification in the field is potentially slowing down the development of clinically meaningful devices in neuropsychiatric healthcare. Still, the advancements made thus far should not be undersold and are impactful. The WBSs discussed herein are demonstrative of analytically sensitive platforms capable of sensing minute changes in peripheral biomarkers with time in complex media. As such, they act as guides to developing more advanced WBSs that could one day lead to a significant paradigm shift in neuropsychiatric healthcare.

## Conflict of Interest

The authors declare no conflict of interest.
